# Cost-Effectiveness of 12 First-Line Treatments for Patients With Advanced EGFR Mutated NSCLC in the United Kingdom and China

**DOI:** 10.3389/fonc.2022.819674

**Published:** 2022-06-06

**Authors:** Haijing Guan, Chunping Wang, Chen Chen, Sheng Han, Zhigang Zhao

**Affiliations:** ^1^Department of Pharmacy, Beijing Tiantan Hospital, Capital Medical University, Beijing, China; ^2^China Center for Health Economic Research, Peking University, Beijing, China; ^3^International Research Center for Medicinal Administration, Peking University, Beijing, China; ^4^Department of Global Health, School of Public Health, Wuhan University, Wuhan, China

**Keywords:** cost-effectiveness, non-small cell lung cancer, epidermal growth factor receptor, the United Kingdom, China

## Abstract

**Background:**

Lung cancer is imposing significant pressure on the national health insurance system worldwide, especially under the COVID-19 pandemic. However, the cost-effectiveness of all available first-line treatments for patients with advanced epidermal growth factor receptor (EGFR) mutated non-small cell lung cancer (NSCLC) is still uncertain. The aim of this study was to evaluate the cost-effectiveness of 12 first-line treatments for patients with advanced EGFR mutated NSCLC from the perspective of the United Kingdom (UK) National Health Service and Chinese health care system.

**Methods:**

We used a Markov model to estimate the cost-effectiveness of 12 treatments, including 6 EGFR tyrosine kinase inhibitors, 4 combination treatments and 2 chemotherapies. The key clinical efficacy and safety data were from a network meta-analysis. The cost and health preference were mainly collected from the literature. The most cost-effective treatment was inferred through a sequential analysis. Uncertainty was tested with one-way sensitivity analyses, scenario analyses, and probabilistic sensitivity analyses. Quality-adjusted life years (QALYs), direct medical costs, and incremental cost-effectiveness ratio (ICER) were estimated, at willingness-to-pay thresholds of £20000 to £50000 and £8000 to £24000 per QALY in the UK and China respectively.

**Results:**

For clinical effectiveness, osimertinib and gefitinib plus pemetrexed based chemotherapy (PbCT) yielded the highest QALYs, while two chemotherapy treatments gained the lowest QALYs. For costs, gefitinib treatment was the cheapest option in both countries (£24529 in the UK and £12961 in China). For cost-effectiveness, 4 treatments including gefitinib, gefitinib plus pemetrexed, gefitinib plus PbCT, and osimertinib formed the cost-effectiveness frontier in both countries. Gefitinib alone (70.7% and 80.0% under the threshold of £20000 and £8000 per QALY in the UK and China, respectively) and gefitinib plus PbCT (62.3% and 71.2% under the threshold of £50000 and £24000 per QALY in the UK and China, respectively) were most likely to be cost-effective compared with other first-line treatments.

**Conclusions:**

Gefitinib and gefitinib plus PbCT were likely to be cost-effective for patients with advanced EGFR mutated NSCLC in both countries.

## Introduction

Lung cancer is one of the most commonly diagnosed cancers worldwide and remains the leading cause of cancer-related deaths (1). Under the COVID-19 pandemic, lung cancer might be one of the worst affected cancers (2). Since much more health care and social resources were used for the COVID-19 pandemic, it was inevitable for decision-makers to choose more cost-effective anti-cancer treatments for lung cancer patients to achieve optimal health resource allocation and affordability of the payers.

Non-small cell lung cancer (NSCLC) accounted for about 85% of lung cancer cases. Somatic activating mutations in epidermal growth factor receptor (EGFR) have been found in approximately 20% of patients with advanced NSCLC worldwide (3), and in 30~50% of Asian patients (4). Over the last decade, EGFR tyrosine kinase inhibitors (TKIs) have been considered the standard first-line treatment of advanced EGFR mutated NSCLC (5). In consideration of potential acquired resistance of EGFR-TKIs, combination strategies of EGFR-TKIs with other treatments in different mechanisms of action have been investigated as potential first-line options (6).

A recently published network meta-analysis (NMA) compared the efficacy and safety of all available first-line treatments for patients with advanced EGFR mutated NSCLC (7). However, it is still uncertain which treatment is cost-effective in all these first-line treatments. Most published economic evaluations have just compared two treatments in the first-line setting for advanced EGFR mutated NSCLC (8–13). Only a few articles compared the cost-effectiveness of multiple treatments for these patients (14–19), but they have not incorporated all available EGFR-TKIs and recent alternative combination treatments, such as gefitinib plus pemetrexed based chemotherapy (PbCT), erlotinib plus bevacizumab et al. Thus, they failed to show the full picture of the cost-effectiveness of all available first-line treatments for advanced EGFR mutated NSCLC.

The objective of this study is to comprehensively analyze the cost-effectiveness of the 12 first-line treatments including 6 EGFR-TKIs (osimertinib, dacomitinib, afatinib, erlotinib, gefitinib, icotinib), 4 combination treatments (afatinib plus cetuximab, erlotinib plus bevacizumab, gefitinib plus PbCT, and gefitinib plus pemetrexed) and 2 chemotherapies (PbCT, pemetrexed free chemotherapy (PfCT)) for patients with advanced EGFR mutated NSCLC. These treatments included both the drugs recommended by clinical guidelines and some combination regimens recently explored in clinical trials, which would provide a more comprehensive picture of the cost-effectiveness of all available treatment options. In addition, since each country’s health care system and drug prices are different, economic evaluations are carried out based on specific countries or regions. To improve the generalizability of this study, the perspectives of the UK National Health Service (NHS) and the Chinese health care system were adopted, as both countries have public single-payment systems, and were typical developed and developing countries in the world respectively.

## Materials and Methods

### Analytical Overview and Model Structure

A Markov model was constructed to estimate the clinical and economic outcomes of 12 first-line treatments for a hypothetical cohort of patients with EGFR mutated NSCLC patients. The model consisted of three exclusive health states: progression-free (PF), progression-disease (PD), and death, as shown in **supplementary efigure 1**. The Markov cycle length was 1 week, which was consistent with other economic evaluations of EGFR-TKIs (4, 18, 20), and the time horizon was lifetime. The model outcomes included life years (LYs), quality-adjusted life years (QALYs) and costs, with cost-effectiveness assessed through the estimation of incremental cost-effectiveness ratio (ICER). All health outcomes and costs were discounted at 3.5% (21) in the UK and 5% (22) in China per year in line with the NICE and Chinese reference case.

The most cost-effective treatment was inferred through a sequential analysis based on the cost-effectiveness frontier. As all treatments in this study were end-of-life anti-cancer drugs, lower and higher boundaries of willingness-to-pay (WTP) threshold in the UK were set to £20000 and £50000 per QALY respectively based on NICE’s recommendation (23, 24). Approximately 1 time (£8000) and 3 times (£24000) the annual gross domestic product (GDP) per capita was used as the lower and higher boundaries of the threshold in China (22). This economic evaluation was based on a literature review and modelling techniques and did not require approval by the institutional research ethics board.

### Clinical Inputs

The clinical data inputs of 12 treatments were derived from the recently published NMA. The virtual patient-level of progression-free survival (PFS) and overall survival (OS) data of the target patients was generated following the standard statistical methodology described by Guyot et al. (25). PfCT, one of the most commonly used comparators in the head-to-head clinical trial of EGFR-TKIs, was set as the reference treatment. The pooled Kaplan-Meier survival curves of PFS and OS of PfCT in these trials were generated consistently with the method in another study (26) (see **supplementary efigures 2, 3**).

To estimate the lifetime health outcome, standard parametric survival models were fitted, including exponential, Weibull, Gompertz, log-logistic and log-normal models. Based on visual fit, statistical goodness-of-fit [Bayesian information criterion (BIC) and Akaike’s information criterion (AIC)], and clinical rationality, the Weibull distribution was chosen for PFS and OS of the PfCT arm (**supplementary efigures 4, 5** and **etables 1, 2**). The time-dependent transition probability at every week was calculated using the survival function of the Weibull distribution:


$$\text{S}\left(\text{t}\right)\;=\;e^{\;\left(-\lambda t^{\gamma }\right)}$$

Where λ is the scale, γ is the shape parameter for the Weibull distribution, and t is the time. The estimated λ and γ were shown in **Table 1**.

**Table 1:** Key clinical inputs.

Parameters	Expected Values	Ranges	Distributions
Weibull parameters of progression-free survival for PfCT			
Scale	0.0474	(0.0359, 0.0625)	Cholesky matrix
Shape	1.5590	(1.4404, 1.6874)	Cholesky matrix
Weibull parameters of overall survival for PfCT			
Scale	0.0075	(0.0049, 0.0114)	Cholesky matrix
Shape	1.3601	(1.2449, 1.4860)	Cholesky matrix
HR of progression-free survival in comparison with PfCT			
Gefitinib	0.37	(0.31, 0.43)	Lognormal (-0.99, 0.08)
Osimertinib	0.16	(0.13, 0.20)	Lognormal (-1.83, 0.11)
Dacomitinib	0.22	(0.16, 0.29)	Lognormal (-1.51, 0.15)
Afatinib	0.31	(0.25, 0.38)	Lognormal (-1.17, 0.11)
Erlotinib	0.33	(0.28, 0.40)	Lognormal (-1.11, 0.09)
Icotinib	0.41	(0.26, 0.66)	Lognormal (-0.89, 0.24)
Afatinib+Cetuximab	0.36	(0.23, 0.57)	Lognormal (-1.02, 0.23)
Erlotinib+Bevacizumab	0.19	(0.14, 0.27)	Lognormal (-1.66, 0.17)
Gefitinib+Pemetrexed	0.25	(0.17, 0.34)	Lognormal (-1.39, 0.18)
Gefitinib+PbCT	0.17	(0.13, 0.22)	Lognormal (-1.77, 0.13)
PbCT	0.68	(0.50, 0.91)	Lognormal (-0.39, 0.15)
HR of overall survival in comparison with PfCT			
Gefitinib	1.02	(0.86, 1.22)	Lognormal (0.02, 0.09)
Osimertinib	0.65	(0.49, 0.85)	Lognormal (-0.43, 0.14)
Dacomitinib	0.78	(0.56, 1.09)	Lognormal (-0.25, 0.17)
Afatinib	0.85	(0.70, 1.05)	Lognormal (-0.16, 0.10)
Erlotinib	1.03	(0.85, 1.25)	Lognormal (0.03, 0.10)
Icotinib	1.08	(0.70, 1.64)	Lognormal (0.07, 0.22)
Afatinib+Cetuximab	1.04	(0.51, 2.17)	Lognormal (0.04, 0.37)
Erlotinib+Bevacizumab	0.83	(0.53, 1.32)	Lognormal (-0.18, 0.23)
Gefitinib+Pemetrexed	0.78	(0.50, 1.27)	Lognormal (-0.25, 0.24)
Gefitinib+PbCT	0.61	(0.45, 0.83)	Lognormal (-0.49, 0.15)
PbCT	1.11	(0.82, 1.49)	Lognormal (0.11, 0.15)

PfCT, pemetrexed free chemotherapy; PbCT, pemetrexed based chemotherapy; HR, hazard ratio.

The ranges are the reported or estimated 95% confidence intervals; the hazard ratios were obtained from the network meta-analysis.

The extrapolated survival curves of PFS and OS for alternative treatments were derived from the published NMA using the hazard ratios (HRs) reported (**Table 1**). Therefore, the Weibull parameter γ for alternative treatments was set equal to γ for pemetrexed free chemotherapy, and the Weibull parameter λ for alternative treatments was calculated as λ for pemetrexed free chemotherapy multiplied by the HR between two treatments. The mortality rate in the PF state was derived from the age-related mortality rate for the general population from the UK and Chinese life tables (27, 28). The elevated mortality rate in clinical trials was assumed to be applied in the PD state.

As the different toxicity spectrums of 12 first-line treatments were shown in the NMA, the costs and disutilities associated with grade 3/4 serious adverse events (SAEs) were considered in this model. The incidence of SAEs was calculated from clinical trials included in the NMA (**supplementary etable 3**), and SAEs with a large impact on medical costs or quality of life were incorporated into our model. Therefore, the following SAEs were considered, including neutropenia, hypertension, rash, anaemia, diarrhoea, leukopenia, thrombocytopenia, dermatitis, fatigue, nausea/vomiting, hair loss and febrile neutropenia.

### Cost and Healthcare Resource Utilization

The direct medical costs considered in this model included drug acquisition costs, drug administration costs, disease management costs, terminal care costs and management costs of SAEs (**Table 2**). The costs were presented in the UK pounds (£ 1 = CNY 8.78), and all costs were adjusted to 2019 prices with the UK (40) and Chinese (41) consumer price index of medical care, respectively.

**Table 2:** Key cost and utility inputs.

Parameters	The UK			China		
	Expected Values (Ranges)	Distributions	References	Expected Values (Ranges)	Distributions	References
Acquisition costs of treatment regimen (£, per cycle)						
Gefitinib	66 (53, 79)	Gamma (96.04, 0.69)	eMIT	33 (27, 40)	Gamma (96.04, 0.35)	Local price
Osimertinib	1346 (1077, 1616)	Gamma (96.04, 14.02)	BNF	146 (117, 175)	Gamma (96.04, 1.52)	Local price;
Dacomitinib	1892 (1514, 2271)	Gamma (96.04, 19.70)	BNF	445 (356, 534)	Gamma (96.04, 4.63)	Local price
Afatinib	506 (405, 607)	Gamma (96.04, 5.27)	BNF	159 (128, 191)	Gamma (96.04, 1.66)	Local price
Erlotinib	121 (97, 145)	Gamma (96.04, 1.26)	eMIT	155 (124, 187)	Gamma (96.04, 1.62)	Local price
Icotinib	—	—	—	153 (122, 184)	Gamma (96.04, 1.59)	Local price
Afatinib+Cetuximab	1303 (1042, 1563)	Gamma (96.04, 13.57)	BNF	794 (635, 952)	Gamma (96.04, 8.26)	Local price
Erlotinib+Bevacizumab	999 (799, 1198)	Gamma (96.04, 10.40)	BNF	608 (486, 729)	Gamma (96.04, 6.33)	Local price
Gefitinib+Pemetrexed	510 (408, 612)	Gamma (96.04, 5.31)	BNF	213 (171, 256)	Gamma (96.04, 2.22)	Local price
Gefitinib+PbCT	526 (421, 631)	Gamma (96.04, 5.48)	eMIT; BNF	240 (192, 288)	Gamma (96.04, 2.50)	Local price
PbCT	453 (362, 543)	Gamma (96.04, 4.71)	eMIT; BNF	224 (179, 269)	Gamma (96.04, 2.34)	Local price
PfCT	24 (19, 29)	Gamma (96.04, 0.25)	eMIT	98 (79, 118)	Gamma (96.04, 1.03)	Local price
Cisplatin+Pemetrexed	449 (359, 538)	Gamma (96.04, 4.67)	eMIT; BNF	185 (148, 222)	Gamma (96.04, 1.93)	Local price
Docetaxel	5 (4, 6)	Gamma (96.04, 0.05)	eMIT	92 (73, 110)	Gamma (96.04, 0.95)	Local price
Administration costs of TKI (£, per cycle)	3 (2, 3)	Gamma (96.04, 0.03)	(20); NHS reference cost	—	—	—
Administration costs of CT/McAb (£, per cycle)	102 (82, 122)	Gamma (96.04, 1.06)	(20); NHS reference cost	13 (12, 15)	Gamma (96.04, 0.14)	(29)
Disease management costs of PFS (£, per cycle)	52 (42, 63)	Gamma (96.04, 0.55)	(20); PSSRU	15 (12, 17)	Gamma (96.04, 0.15)	(11)
Disease management costs of PD (£, per cycle)	55 (44, 66)	Gamma (96.04, 0.57)	(20); PSSRU	15 (12, 17)	Gamma (96.04, 0.15)	(11)
BSC cost (£, per cycle)	100 (80, 120)	Gamma (96.04, 1.04)	(30)	88 (71, 106)	Gamma (96.04, 0.92)	(11)
Terminal care cost (£)	4576 (3660, 5491)	Gamma (96.04, 47.64)	(20); PSSRU	1880 (1504, 2256)	Gamma (96.04, 19.57)	(31)
Management costs of adverse events (£, per event)						
Diarrhoea	1241 (993, 1490)	Gamma (96.04, 12.92)	(30)	4 (4, 5)	Gamma (96.04, 0.05)	(31)
Fatigue	2638 (2111, 3166)	Gamma (96.04, 27.47)	(30)	99 (79, 119)	Gamma (96.04, 1.03)	(31)
Febrile neutropenia	11687 (9350, 14025)	Gamma (96.04, 121.69)	(30)	869 (695, 1043)	Gamma (96.04, 9.05)	(16)
Nausea/Vomiting	1241 (993, 1490)	Gamma (96.04, 12.92)	(30)	54 (43, 65)	Gamma (96.04, 0.56)	(31)
Neutropenia	2048 (1638, 2457)	Gamma (96.04, 21.32)	(30)	396 (317, 475)	Gamma (96.04, 4.12)	(31)
Rash	130 (104, 156)	Gamma (96.04, 1.35)	(30)	4 (4, 5)	Gamma (96.04, 0.05)	(31)
Hypertension	2212 (1770, 2655)	Gamma (96.04, 23.03)	(32)	10 (8, 12)	Gamma (96.04, 0.11)	(11)
Leukopenia	322 (258, 386)	Gamma (96.04, 3.35)	(33)	82 (66, 99)	Gamma (96.04, 0.86)	(31)
Anaemia	796 (637, 955)	Gamma (96.04, 8.29)	(30)	456 (365, 547)	Gamma (96.04, 4.75)	(31)
Dermatitis	130 (104, 156)	Gamma (96.04, 1.35)	Assumption	4 (4, 5)	Gamma (96.04, 0.05)	Assumption
Thrombocytopenia	327 (262, 392)	Gamma (96.04, 3.4)	(34)	416 (333, 499)	Gamma (96.04, 4.33)	(31)
Hair loss	0	Fixed	(35)	0	Fixed	(35)
Utilities of health states						
PFS	0.883(0.71, 1.00)	Beta (15.38, 2.04)	(36)	0.815 (0.65, 0.98)	Beta (16.95, 3.85)	(36)
PD	0.166 (0.13, 0.20)	Beta (79.93, 401.58)	(36)	0.321 (0.26, 0.39)	Beta (64.89, 137.26)	(36)
Body surface area (m^2^)	1.79 (1.43, 2.15)	Normal (1.79, 0.18)	(37)	1.72 (1.5, 1.9)	Normal (1.72, 0.1)	(16)
Weight (kg)	75 (60, 90)	Normal (75, 7.65)	Assumption	65 (52, 78)	Normal (65, 6.63)	(16)
Age of newly-diagnosed advanced NSCLC	71.4	Fixed	(38)	61.6	Fixed	(39)
Discount rate	3.5% (0, 6%)	Uniform		5% (0, 8%)	Uniform	

PfCT, pemetrexed-free chemotherapy; PbCT, pemetrexed based chemotherapy; TKI, tyrosine kinase inhibitors; CT, chemotherapy; McAb, monoclonal antibody; PFS, progression-free survival; OS, overall survival; BSC, best support care; NSCLC, non-small cell lung cancer; eMIT, the electronic market information tool 2018/2019; BNF, the British national formulary; PSSRU, Personal Social Services Research Unit 2020.

Assuming the costs of the demnatitis is the same as the rash. Osimertinib has been applied for reimbursement for the first-line treatment in 2020 NRDL re-negotiation, which will lead to further high price-cut according to NHSA’s guidance. However, because osimertinib has applied for keeping the price confidential, we assumed that the price-cut of osimertinib was equal to almonertinib (one of the third-generation EGFR TKIs) with a 64.08% drop.

EGFR TKIs were administered until disease progression or death, which were osimertinib at a dose of 80 mg once a day, dacomitinib at the dose of 45 mg once a day, afatinib at a dose of 40 mg once a day, erlotinib at a dose of 150 mg once a day, icotinib at a dose of 125 mg three times a day and gefitinib at a dose of 250 mg once a day. Chemotherapies were administered every 3 weeks up to 4 chemotherapy cycles. The combination treatments were administered depending on the specific regimen they contained according to the clinical trials. The detailed administration information of 12 first-line treatments was shown in **supplementary etable 4**. To calculate the mean dosages of chemotherapies per cycle, an average body surface area of 1.79 m^2^ (37) and 1.72 m^2^ (15) were assumed for the UK and Chinese patients in the base-case analysis (**Table 2**). We assumed no vial-sharing in this analysis as the practice of vial-sharing was still very limited in clinical practice in both countries.

After disease progression, 61.0% and 52.8% of patients would receive second-line active anti-cancer treatments in the UK (30) and China (42), respectively, and the remaining patients would receive the best supportive care (BSC) directly. The subsequent treatments of each arm were elicited from NICE Clinical Guidance NG122 and Medical Association guidelines for clinical diagnosis and treatment of lung cancer (Edition 2018) in the UK (43) and China (44), respectively. Patients in the EGFR TKIs arms alone or together with monoclonal antibodies would receive second-line pemetrexed plus platinum for up to four chemotherapy cycles, third-line docetaxel, and followed by BSC; while patients in the chemotherapy or gefitinib combined chemotherapy arms would receive second-line docetaxel, followed by BSC. The clinical pathways of each arm were shown in **supplementary efigure 1**.

For the UK setting, the drug acquisition costs were obtained from the electronic market information tool (eMIT) (45) if available, otherwise were obtained from the British national formulary (BNF) (46). The healthcare resource utilization data was derived from the published HTA reports for untreated advanced EGFR mutated NSCLC (20, 30, 35, 47). Unit costs were sourced from NHS Reference Costs 2018/2019 (48) and the Personal and Social Services Research Unit 2020 (49). The terminal care cost was based on the data from previous NSCLC HTA reports (20, 30, 49). As icotinib has not been approved in the UK at the time of this study, it was not considered in the UK setting.

For the Chinese setting, the acquisition costs of the drug were either derived from the documents of National Reimbursement Drug List (NRDL) negotiation or Volume-based Procurement (VBP) by the National Healthcare Security Administration (NHSA) if available, or derived from YAOZHI database, which collects updated drug prices around the country. Dacomitinib was not covered by the NRDL at the time of this study, thus the acquisition cost of dacomitinib was estimated on the latest retail price and was adjusted according to the patient assistance program (PAP). The drug administration costs and disease management costs were obtained from published studies as with previous economic evaluations in China (11, 29).

### Health Utilities

Health utility values of the PF and PD states were adopted from a recent international study in the base case analysis, which captured health utilities in the UK, China, and other countries or regions (36). The health utilities for the PF and PD state were 0.883 and 0.166 in the UK and were 0.815 and 0.321 in China. The disutilities of 12 kinds of SAEs were also obtained from previously published studies (15, 31, 36).

### Sensitivity Analyses

In consideration of the uncertainty of model parameters and assumptions, deterministic sensitivity analysis (DSA) and probabilistic sensitivity analysis (PSA) were conducted to evaluate the robustness of the base case results. DSA included one-way sensitivity analyses and scenario analyses.

In the one-way sensitivity analyses, parameters were independently varied within a plausible range determined by either published data or by 95% confidence intervals. If not applicable, the values were varied by ± 20% of the corresponding base case value.

The following scenario analyses were also performed. For the clinical efficacy of osimertinib, the analysis was conducted based on the updated OS data from the FLAURA clinical trial for advanced EGFR mutated NSCLC (50). For health utilities of the PF and PD states, the analysis was conducted based on Nafees et al.’s study (51) and Shen et al.’s study (52) for the UK and Chinese patients, respectively. For the drug acquisition costs, the analysis was carried out using the BNF prices in the UK. For the subsequent treatments, it was assumed that approximately 60% (53) of patients would develop the T790M mutation when treated with first- or second-generation EGFR TKIs, these patients would receive osimertinib as second-line treatments, and other patients were assumed to receive PbCT.

The PSA was conducted using second-order Monte Carlo simulation by running 5000 iterations to account for uncertainty in model parameters. Gamma distributions were used for costs, lognormal distribution for HR parameters, and beta distributions were used for utilities, proportions and probabilities (54). The scatter plots in the cost-effectiveness plane were conducted to show the distribution of ICERs for the treatments on the cost-effectiveness frontier, and cost-effectiveness acceptability curves (CEACs) were considered to show the probabilities of each treatment being cost-effective at a wide range of WTP thresholds.

## Results

### Base-Case Results

For health outcomes, the range of QALYs for 12 treatments were from 0.744 to 1.762 and from 1.061 to 1.935 in the UK and China, respectively. The pemetrexed free chemotherapy yielded the lowest QALYs (0.744 and 1.061 in the UK and China), followed by pemetrexed based chemotherapy (0.861 and 1.101); while osimertinib gained the highest QALYs (1.762 and 1.935), followed by gefitinib plus pemetrexed based chemotherapy (1.687 and 1.918) in both the UK and China (**Table 3**). The pemetrexed free chemotherapy (-0.071 and -0.039) suffered from the worst QALYs loss associated with SAEs in both countries, followed by gefitinib plus pemetrexed based chemotherapy (-0.059 and -0.036). Osimertinib (-0.003) and icotinib (-0.000) had the lowest QALYs loss associated with SAEs in the UK and China, respectively (see **supplementary etables 5, 6**).

**Table 3:** Base case results of cost-effectiveness for 12 first-line treatments in the UK and China.

**Treatments**	**The UK**					**China**				
	**Costs (£)**	**QALYs**	**LYs**	**ICER** ^a^ **(£/QALY)**	**sequential ICER** ^b^ **(£/QALY)**	**Costs (£)**	**QALYs**	**LYs**	**ICER** ^a^ **(£/QALY)**	**sequential ICER** ^b^ **(£/QALY)**
Gefitinib	24529	1.130	2.571	-1217	Dominant	12961	1.312	2.572	-7266	Dominant
Osimertinib	139483	1.762	3.485	112412	1269085	25459	1.935	3.485	12215	224999
Afatinib	51865	1.255	2.899	52570	Dominated	21478	1.469	2.900	16418	Dominated
Icotinib^c^	—	—	—	—	—	18308	1.254	2.482	18310	Dominated
Dacomitinib	155510	1.475	3.080	178439	Dominated	22517	1.656	3.081	13009	Dominated
Erlotinib	27237	1.177	2.554	5169	Extended dominated	19270	1.340	2.554	16118	Dominated
Afatinib+Cetuximab	92935	1.113	2.535	184107	Dominated	52380	1.297	2.536	159297	Dominated
Erlotinib+Bevacizumab	106486	1.541	2.950	102246	Dominated	58504	1.673	2.951	71527	Dominated
Gefitinib+Pemetrexed	33221	1.401	3.080	12513	32144	16873	1.606	3.081	3842	13289
Gefitinib+PbCT	44445	1.687	3.632	20609	39175	21545	1.918	3.633	7895	14966
PbCT	31595	0.861	2.426	56160	Dominated	16066	1.101	2.427	32121	Dominated
PfCT	24999	0.744	2.607	Reference	Dominated	14780	1.061	2.607	Reference	Dominated

PfCT, pemetrexed free chemotherapy; PbCT, pemetrexed based chemotherapy; QALY, quality-adjusted life year; LY, life year; ICER, incremental cost-effectiveness ratio.

a compared with PfCT;

b sequential ICER was compared with the next best non-dominated option;

c icotinib was not approved in the UK at the time of this study.

For costs, some significant differences were shown between the UK and China. In the UK, gefitinib treatment was the lowest-cost option (£24529), followed by pemetrexed free chemotherapy (£24999) and erlotinib (£27237), all of which were lower than £30000. Dacomitinib (£155510) and osimertinib (£139483) yielded the highest cost, followed by erlotinib plus bevacizumab (£106486), all of which were higher than £100000. In China, gefitinib was also the cheapest treatment (£12961), followed by pemetrexed free chemotherapy (£14780) and pemetrexed based chemotherapy (£16066). Erlotinib plus bevacizumab (£58504) and afatinib plus cetuximab (£52380) were yielded with the highest costs, followed by osimertinib (£25459).

**Figure 1:**
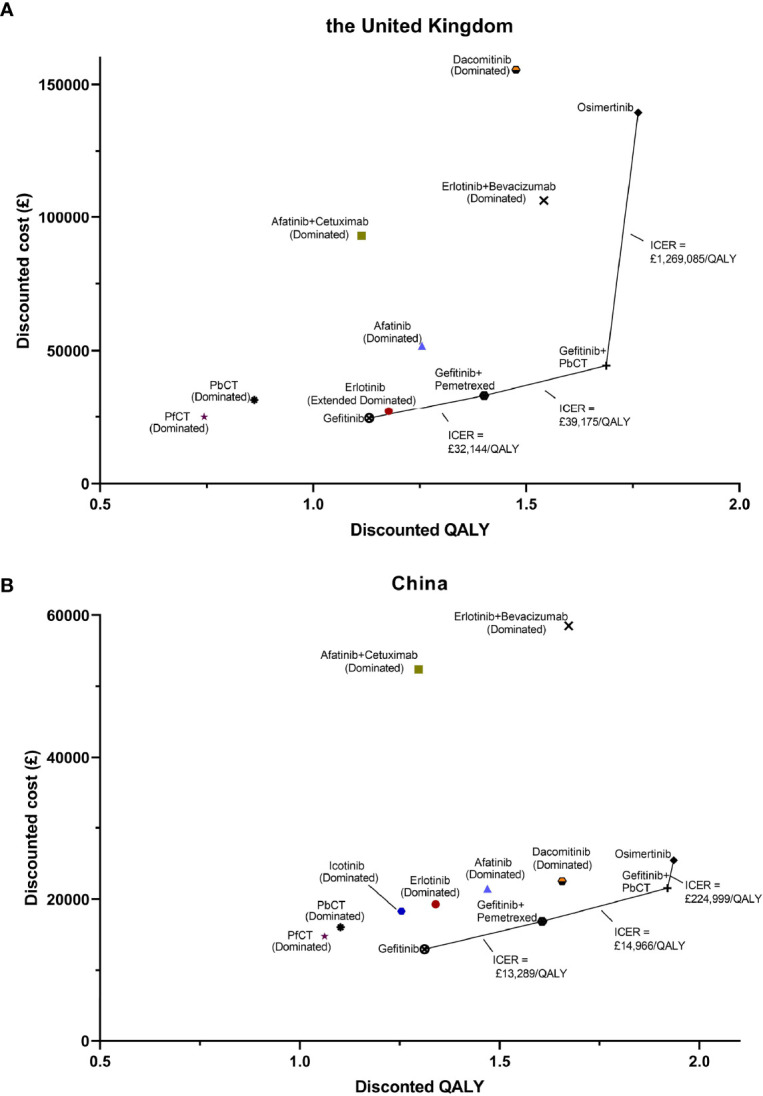
Cost-effective frontier of the 12 first-line treatments for patients with advanced epidermal growth factor receptor mutated non-small lung cancer in the UK setting **(A)** and the Chinese setting **(B)**. (PfCT, pemetrexed free chemotherapy; PbCT, pemetrexed based chemotherapy; QALY, quality-adjusted life year).

For cost-effectiveness, gefitinib, gefitinib plus pemetrexed, gefitinib plus pemetrexed based chemotherapy, and osimertinib formed the cost-effectiveness frontier in both the UK and China, which indicated that just these four treatments might be cost-effective, while other treatments were either dominated or subject to extend dominance, shown in **Figure 1**. By virtue of its lower costs and greater QALYs, the cheapest gefitinib treatment dominated both pemetrexed free chemotherapy and pemetrexed based chemotherapy in the UK and China. The sequential ICERs of gefitinib plus pemetrexed versus gefitinib, gefitinib plus pemetrexed based chemotherapy versus gefitinib plus pemetrexed, and osimertinib versus gefitinib plus pemetrexed based chemotherapy were £32144/QALY, £39175/QALY, and £1269085/QALY in the UK, while whose were £13289/QALY, £14966/QALY, and £224999/QALY in China.

### Sensitivity Analyses Results

The results of the one-way sensitivity analyses were shown in **supplementary efigure 6**, which reported the similar expected values for ICERs of treatments that laid on the cost-effectiveness frontier with those in the base-case analysis. For example, in the comparison of gefitinib plus pemetrexed based chemotherapy with pemetrexed free chemotherapy in the UK and Chinese setting, the HR of PFS, the utility of PFS, and the HR of OS were the most influential parameters in the model. The ICERs of both treatments on the basis of lower and upper values of each parameter were all lower than £50000/QALY and £24000/QALY in the UK and China respectively, which were consistent with the base-case results.

The results of scenario analyses also found the results to be relatively robust. When the updated OS data of osimertinib was used in the model, the QALYs were 1.818 and 2.044 in the UK and China respectively, which were 0.056 and 0.108 higher than those in the base-case analysis (**supplementary etables 7, 8**). The updated ICER of osimertinib was lower than the original one (£109060/QALY vs £112412/QALY) in the UK, while it was slightly higher than the original one (£12690/QALY vs £12215/QALY) in China. When health utilities of PF and PD states from the Nafees et al.’s study and Shen et al.’s study were used in the model for the UK and China respectively, the QALYs of each treatment were higher than those in the base-case analysis. The distribution of ICERs of each treatment was similar with that in base-case analysis, except that pemetrexed based chemotherapy was dominated by pemetrexed free chemotherapy in both the UK and China (**supplementary etables 9, 10**). When the disutilities of SAEs originally reported in Nafees et al. were conducted, the updated results of the UK and China settings were very close to those in the base-case analysis (**supplementary etables 11, 12**). When the BNF prices in the UK were applied in the model, the total costs of gefitinib, gefitinib plus pemetrexed and gefitinib plus pemetrexed based chemotherapy were significantly higher than those in the base-case analysis, which made pemetrexed free chemotherapy not dominated by gefitinib (**supplementary etable 13**). When osimertinib was considered for patients with T790M mutation after the failure of first- or second-generation EGFR TKIs monotherapy or with monoclonal antibodies, the total costs of these treatments were slightly increased compared to base-case analysis. The distribution of ICERs of these treatments was consistent with that in the base-case analysis (**supplementary etables 14, 15**).

**Figure 2:**
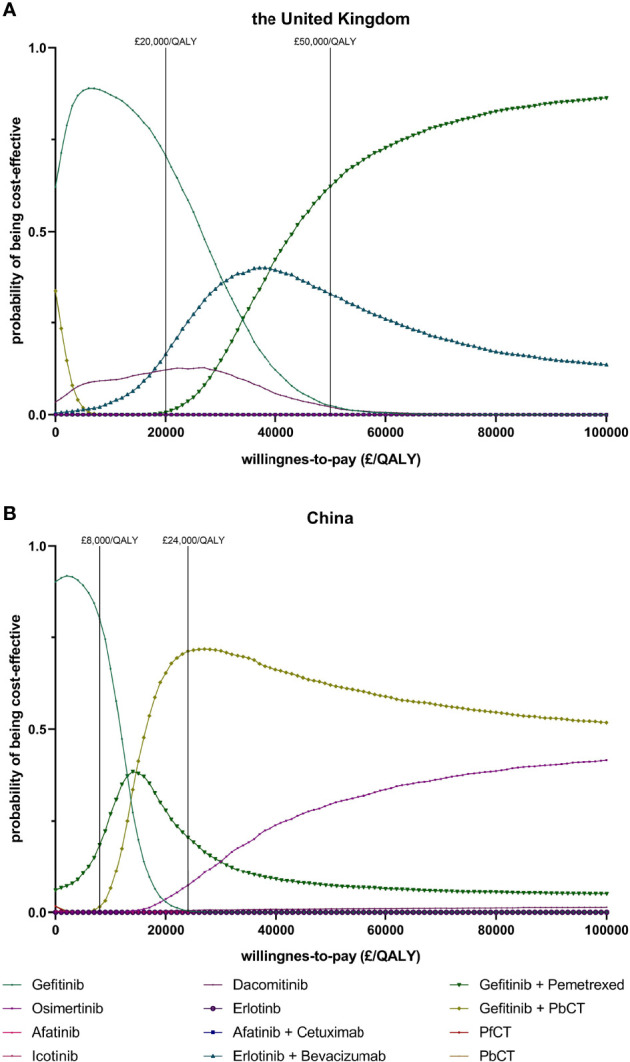
Cost effectiveness acceptability curves indicating the probability of each first-line treatment to be cost-effective at different willingness to pay thresholds in the UK **(A)** and the Chinese setting **(B)**. (PfCT, pemetrexed free chemotherapy; PbCT, pemetrexed based chemotherapy; QALY, quality-adjusted life year).

**Figure 3:**
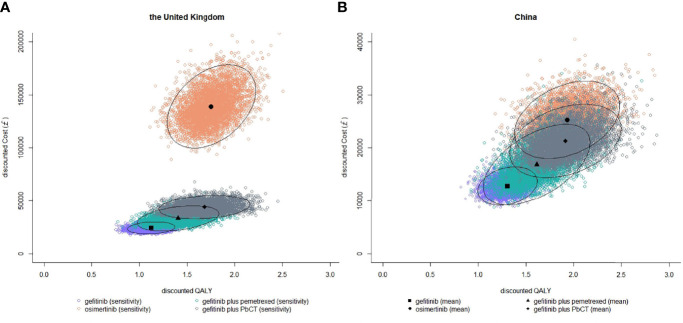
Probabilistic scatter plots of costs and QLAYs of gefitinib, gefitinib plus pemetrexed based chemotherapy, and osimertinib for a cohort of 5000 in the UK **(A)** and the Chinese setting **(B)**. Ellipses surround 95% of estimates.

The results of PSA showed that the probabilities of each treatment being cost-effective at different WTP thresholds (**Figure 2**). In the UK, when the WTP threshold was £20000/QALY, the probability of being cost-effective was highest for gefitinib (70.7%), compared with 16.4% for gefitinib plus pemetrexed and 12.2% for erlotinib. When the WTP threshold was £50000/QALY, the probability of being cost-effective was optimal for gefitinib plus pemetrexed based chemotherapy (62.3%), followed by 33.0% for gefitinib plus pemetrexed and 2.5% for gefitinib. In China, when the WTP threshold was £8000/QALY which was close to the GDP per capita in 2020, the probability of being cost-effective was highest for gefitinib (80.0%), followed by 18.5% for gefitinib plus pemetrexed and 1.5% for gefitinib plus pemetrexed based chemotherapy. When the WTP threshold was £24000/QALY, the probability of being cost-effective was 71.2% for gefitinib plus pemetrexed based chemotherapy, greater than gefitinib plus pemetrexed (20.6%) and osimertinib (7.4%). The scatter plots in **Figure 3** showed the most possible distributions of QALYs and costs for 4 treatments on the cost-effectiveness frontiers, which were also consistent with the basis-case results.

## Discussion

To our knowledge, this is the first study to comprehensively summarize the cost-effectiveness of all available first-line treatments for patients with advanced EGFR mutated NSCLC from the UK and China’s health care system perspective. The principal findings suggested that gefitinib, gefitinib plus pemetrexed, gefitinib plus PbCT, and osimertinib formed the cost-effectiveness frontier in both the UK and China. When the WTP was based on the lower value of commonly used threshold in the UK and China, gefitinib was most likely to be cost-effective. When the threshold increased, gefitinib plus PbCT was most likely to be cost-effective.

The clinical effectiveness and cost-effectiveness showed the different pictures for decision makers if they would like to choose the most valuable treatment. As for clinical effectiveness, simultaneous treatment with gefitinib plus pemetrexed based chemotherapy and gefitinib plus pemetrexed were associated with longer LYs and higher QALYs versus gefitinib alone. Similarly, the combination treatment of erlotinib plus bevacizumab also had better clinical benefit than erlotinib alone. These findings were consistent with those in recent published NMA, which favoured these two combination treatments for the persistent response based on objective response rate (ORR) and the extension of PFS, respectively. Therefore, the results showed that patients with advanced EGFR mutated NSCLC might obtain overall clinical benefits from the addition of pemetrexed based chemotherapy or bevacizumab to the current standard of care. However, the combination use of afatinib plus cetuximab has not yet shown a better clinical benefit than afatinib alone.

As for cost-effectiveness, the total lifetime medical costs of gefitinib plus pemetrexed and gefitinib plus pemetrexed based chemotherapy were only slightly higher than that of gefitinib (£8692 and £19916) in the UK. Gaining much more QALYs with slightly higher costs of both two combination treatments compared to the corresponding EGFR TKI alone, these two combination treatments were more likely to be cost-effective at the threshold of £50000/QALY. However, the total cost of erlotinib plus bevacizumab was £106485 higher than that of erlotinib, which might lead to a substantial financial burden on patients and public payers, and make erlotinib plus bevacizumab not likely to be cost-effective. In other words, combination treatment might improve the clinical benefit, but whether it also improved the cost-effectiveness depending on the ratio of incremental costs to incremental QALYs of two treatments, and the potential change of toxicity spectrums.

The EGFR TKIs were associated with less toxicity, while the combination treatments caused more. The additional SAEs for these combination treatments were reflected in the patients’ quality of life and related medical costs. For instance, higher incidence rates of fatigue (4.76% vs 0.61%), neutropenia (27.14% vs 0.20%), leukopenia (17.14% vs 0.10%), anemia (17.14% vs 0.40%) and thrombocytopenia (13.81% vs 0.00%) were observed with the addition of pemetrexed based chemotherapy to gefitinib. Therefore, QALYs loss associated with SAEs for gefitinib plus pemetrexed based chemotherapy was 0.059 compared with 0.004 for gefitinib alone, while the SAEs-related costs were £1295 and £77 in the UK, respectively. In these 12 first-line treatments, erlotinib plus bevacizumab and icotinib had the worst and best safety profiles on the basis of SAE incidence, respectively. When considering the quality of life and cost associated with SAEs, pemetrexed free chemotherapy suffered from the worst profile, followed by gefitinib plus pemetrexed based chemotherapy; while icotinib had the best profile, followed by osimertinib. For a better treatment selection, knowledge of the main SAEs associated with each treatment during long-term use was critical, because anti-cancer treatment should be based on the individual characteristics of the target patient, not just based on the average results from the studies.

Compared with the recently published economic evaluations investigating multiple treatments for patients with advanced EGFR mutated NSCLC, our present study had several strengths. This study covered all available first-line treatments, including 6 EGFR TKIs, 4 combination treatments, and 2 chemotherapies. This multiple comparison could provide more necessary information than the study comparing just two treatments and would be more helpful to a clinician to choose the optimal option. The efficacy of 3 combination treatments (gefitinib plus pemetrexed based chemotherapy, gefitinib plus pemetrexed, and erlotinib plus bevacizumab) have already been proven significantly better than that of EGFR TKI used alone, and the cost-effectiveness of these combination options became the most crucial question that decision makers concerned about. Moreover, due to the different health care systems, cost-effectiveness analysis was generally carried out separately based on a specific country or region, which resulted in the lack of comparability and external validity of different studies. As both the UK and China were public single-payment systems and were developed and developing countries respectively, the generalizability of these results were much better than other studies. For instance, one of the important findings of this study was that although drug prices were obviously different in the UK and China, gefitinib, gefitinib plus pemetrexed, gefitinib plus pemetrexed based chemotherapy, and osimertinib were on the cost-effectiveness frontier in both countries. This finding also had a high reference value for anti-cancer drug evaluation and selection in other countries or regions worldwide. Our study comprehensively analyzed the incidence of 12 kinds of SAEs for each treatment and their potential effect on the quality of life and cost, which was lacking in other economic evaluations. Although SAEs had a limited impact on the results of cost-effectiveness analysis, they were necessary and helpful for the clinician to promote rational use of anti-cancer drugs. In addition, all available treatments for advanced EGFR mutated NSCLC were considered in our study, thus a single threshold was impossible to represent decision maker’s WTP for QALYs gained and was not appropriate in all decision contexts (55). For such reason, we adopted commonly used higher and lower WTP boundaries in the UK and China to support more flexible decision making.

The overall results in our study were consistent with the published economic evaluations. A research published recently compared the cost-effectiveness of 3 EGFR TKIs (afatinib, gefitinib, and erlotinib) and pemetrexed plus cisplatin in China (15). It found afatinib gained an additional 0.382, 0.216 and 0.174 QALYs in comparison with pemetrexed plus cisplatin, gefitinib and erlotinib, which was close to the expected incremental values in our study (0.368, 0.157, and 0.129 QALYs, respectively). In the other two cost-effectiveness analyses, afatinib instead of gefitinib provided an extra 0.170 QALYs in France (12) and 0.160 QALY in the Netherlands (56), which was also very close to the counterpart in our study. As for the health outcome of afatinib versus erlotinib, both Gu et al. (15) and our study found the QALY of afatinib was higher than that of erlotinib. However, an American study found erlotinib had an incremental 0.11 QALY compared with afatinib (57). The inconsistency of these findings might result from the different sources of clinical data inputs. The incremental QALYs of osimertinib versus gefitinib in our study were 0.632 in the UK and 0.638 in China, which implied that the clinical effectiveness of osimertinib was better than that of gefitinib. This result was consistent with other economic evaluations thought the magnitude varied in different settings. For instance, the incremental QALYs of osimertinib were 0.200 for Spain (58), 0.274 for Australia (19), 0.319 for Singapore (10), 0.790 for Canada (59), 0.550 and 0.594 for US (8, 18), 0.487 and 0.650 for China (8, 60). When considering its high drug cost, osimertinib was not cost-effective compared to gefitinib in all the above studies.

The sensitivity analyses showed that the overall results remained relatively robust. One-way sensitivity analysis of gefitinib plus pemetrexed based chemotherapy versus pemetrexed free chemotherapy showed that the HR of PFS, the utility of PFS, and the HR of OS were the three most influential parameters of the model. When these three parameters were increased or decreased using the upper or lower boundaries, the ICERs were always in the cost-effective range. In addition, the QALYs of base-case analysis in our study was slightly lower than those in other studies. This was because the health utilities of PFS and PD states used in our model were derived from a recent international study, the utility of PD state was 0.166 in this study, which was lower than in other studies. For instance, in Nafees et al.’s former study, the utility of PD state was 0.473. In a review of health state utility values used in UK NICE appraisals in advanced NSCLC, the range of utilities used in PD state was 0.47 to 0.69 (61). Thus, a value of 0.473 was used in our model in the scenario analyses, and the QALY results increased as expected. This adjustment did not change the main results of our study. The reason for using these health utilities from this international study was because it was currently the only study that simultaneously reported the health utilities of advanced lung cancer patients treating in the first-line setting in the UK and China. Using these utilities could improve the internal validity of our study, that is, to ensure that the results of QALYs are comparable between the UK and China.

This study had several limitations. Firstly, because the clinical data simultaneously evaluating all available first-line treatments for advanced EGFR mutated NSCLC in one clinical trial is not available, this economic evaluation was based on a recently published NMA. Fortunately, network transitivity, heterogeneity, and inconsistency were thoroughly investigated by this high-quality NMA. Secondly, indirect costs related to the productivity loss were not covered in our study. However, clinical and health insurance decision makers paid more attention to the direct medical costs, which might directly affect the clinician’s choice of medication and the sustainability of health insurance funding. Thirdly, for the absence of medical cost research that covered the 12 kinds of SAEs in our model, the SAE costs were derived from different published studies. To explore the potential bias and uncertainty, these parameters were all tested in the one-way sensitivity analyses and the results indicated that it had minimal impact on the results.

## Conclusions

In this economic evaluation, the clinical effectiveness and cost-effectiveness of 12 first-line treatments for advanced EGFR mutated NSCLC were evaluated based on the perspective of the UK NHS and the Chinese health care system. For clinical effectiveness, osimertinib and gefitinib plus PbCT yielded the highest QALYs, while two chemotherapy treatments gained the lowest QALYs. When considering costs, gefitinib alone and gefitinib plus PbCT were likely to be the most cost-effective option based on the commonly used threshold in the UK and China. These findings could help decision-makers make a better balance between improving health outcome and saving medical cost, which was very necessary for the context of limited health resources and funding, especially under the COVID-19 pandemic worldwide.

## Data Availability Statement

The original contributions presented in the study are included in the article/**Supplementary Material**. Further inquiries can be directed to the corresponding author.

## Author Contributions

HG designed the study. CW carried out the computer simulations and analysis. HG, CC, SH, and ZZ contributed to critical review and modification. HG drafted the manuscript for all other authors to amend and comment on. ZZ is the guarantor. All authors approved the final version to be published.

## Funding

This study was funded by the National Natural Science Foundation of China, grant no. 72104151.

## Conflict of Interest

The authors declare that the research was conducted in the absence of any commercial or financial relationships that could be construed as a potential conflict of interest.

## Publisher’s Note

All claims expressed in this article are solely those of the authors and do not necessarily represent those of their affiliated organizations, or those of the publisher, the editors and the reviewers. Any product that may be evaluated in this article, or claim that may be made by its manufacturer, is not guaranteed or endorsed by the publisher.
