# Supplementary material for: Cost-Effectiveness of 12 First-Line Treatments for Patients With Advanced EGFR Mutated NSCLC in the United Kingdom and China
Source: Front Oncol. 2022 Jun 6;12:819674. doi: 10.3389/fonc.2022.819674 (PMC9241581; doi:10.3389/fonc.2022.819674)
Supplement: Supplementary file 1 [file DataSheet_1.docx]

**Supplementary Material**

**Cost-Effectiveness of 12 First-Line Treatments for Patients With Advanced EGFR Mutated NSCLC in the United Kingdom and China**

Haijing Guan^1,2^, Chunping Wang^3^, Chen Chen^2,4^, Sheng Han^3^ and Zhigang Zhao^1^*

1 Department of Pharmacy, Beijing Tiantan Hospital, Capital Medical University, Beijing, China, 2 China Center for Health Economic Research, Peking University, Beijing, China, 3 International Research Center for Medicinal Administration, Peking University, Beijing, China, 4 Department of Global Health, School of Public Health, Wuhan University, Wuhan, China


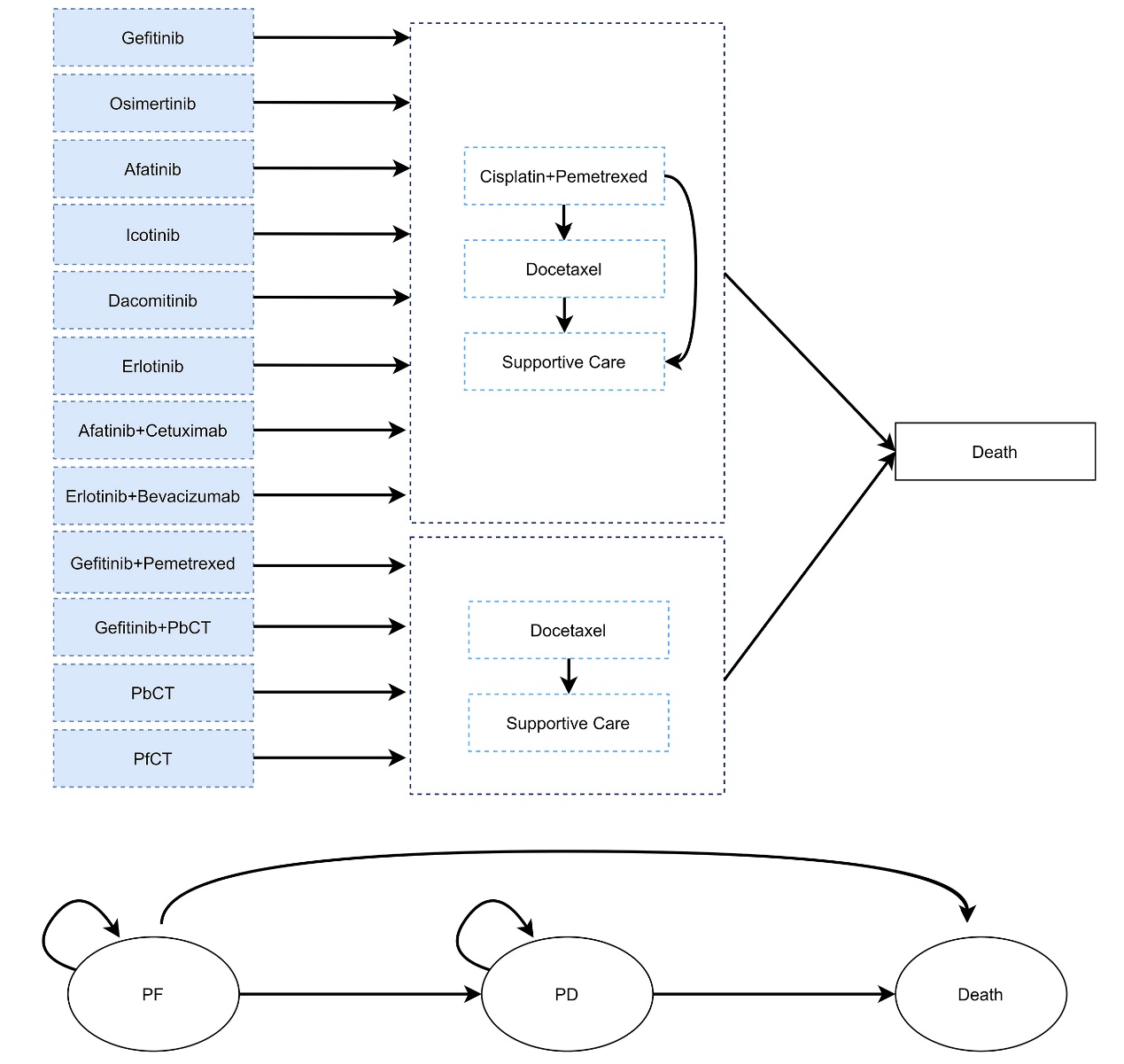


**eFigure 1.** Clinical pathways and Markov model structure.

Abbreviation: PfCT: pemetrexed free chemotherapy; PbCT: pemetrexed based chemotherapy


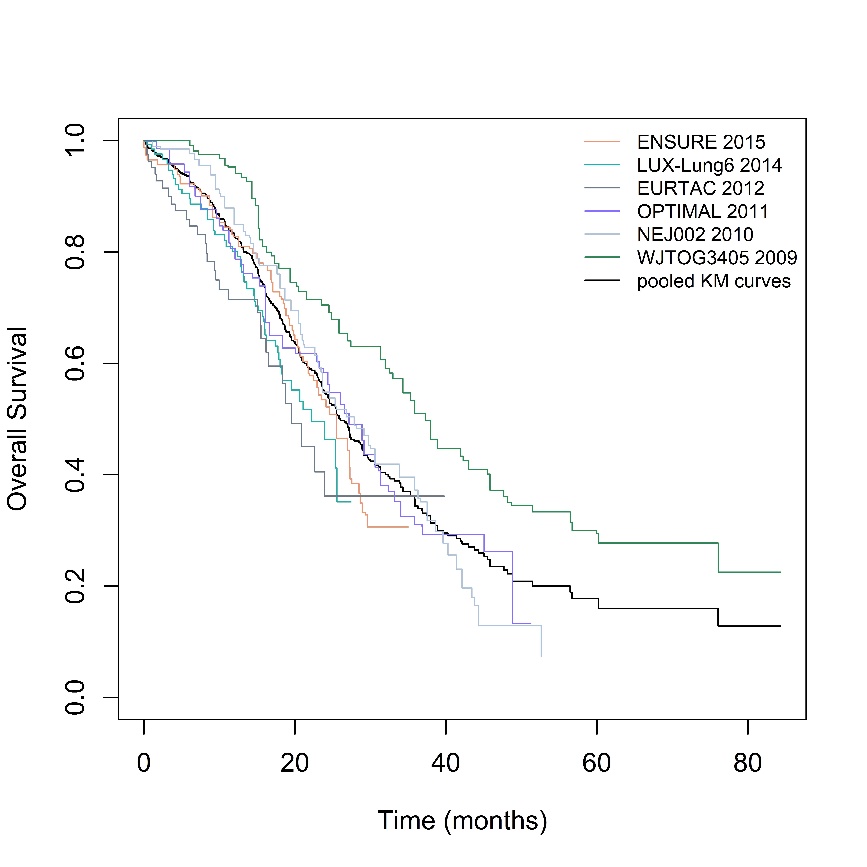


**eFigure 2.** Curves of the overall survival for the untreated patients with advanced non-small cell lung cancer harboring EGFR mutations in the five studies of pemetrexed free chemotherapy arm in network meta-analysis.


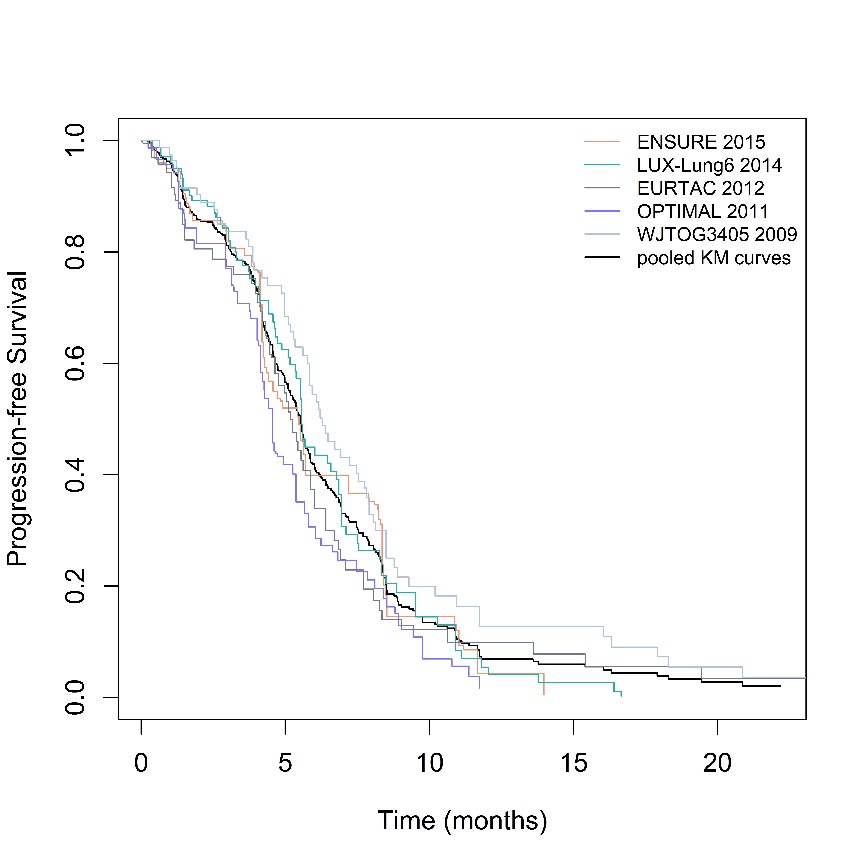


**eFigure 3.** Curves of the progression-free survival for the untreated patients with advanced non-small cell lung cancer harboring EGFR mutations in the five studies of pemetrexed free chemotherapy arm in network meta-analysis.


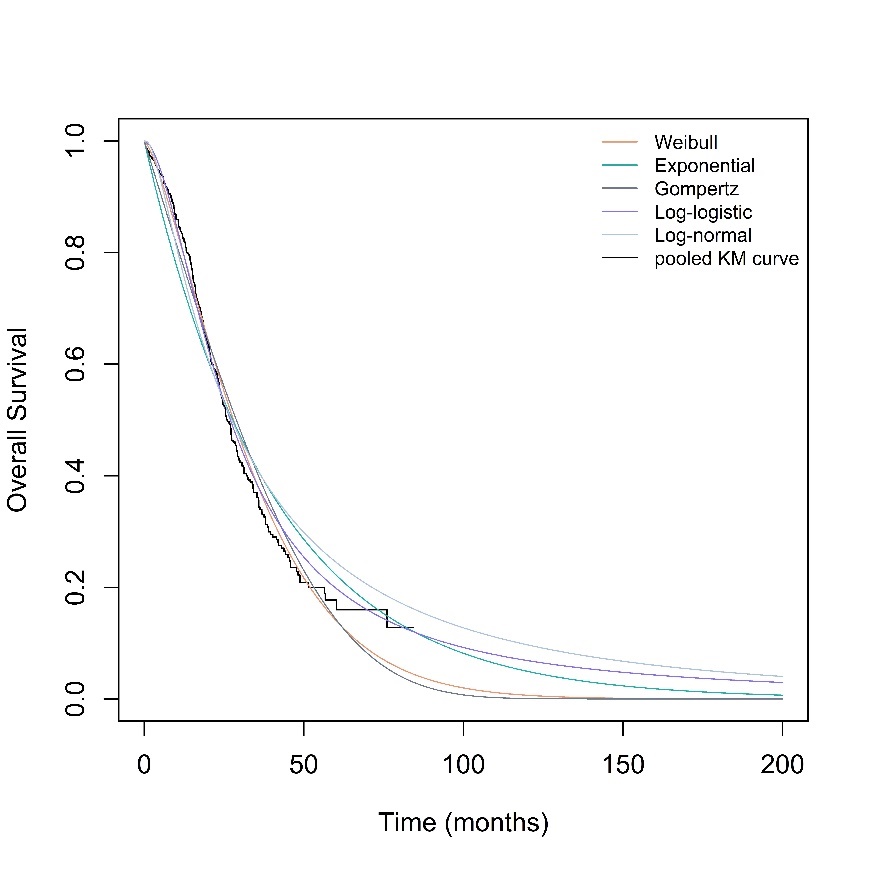


**eFigure 4.** Overall survival Kaplan-Meier curve of pemetrexed free chemotherapy fitting and extrapolation


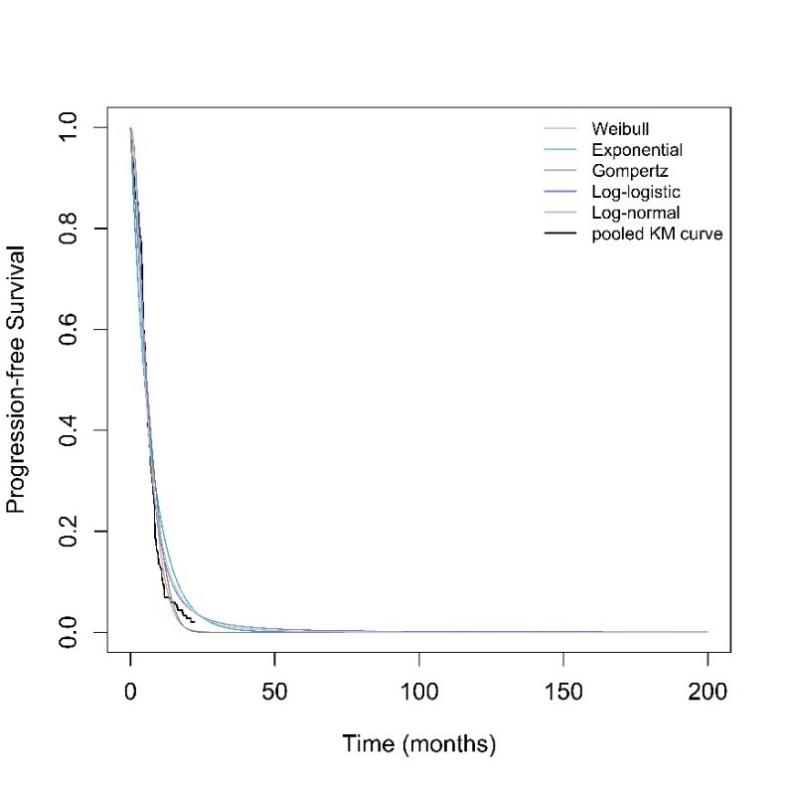


**eFigure 5.** Progression-free survival Kaplan-Meier curve of pemetrexed free chemotherapy fitting and extrapolation


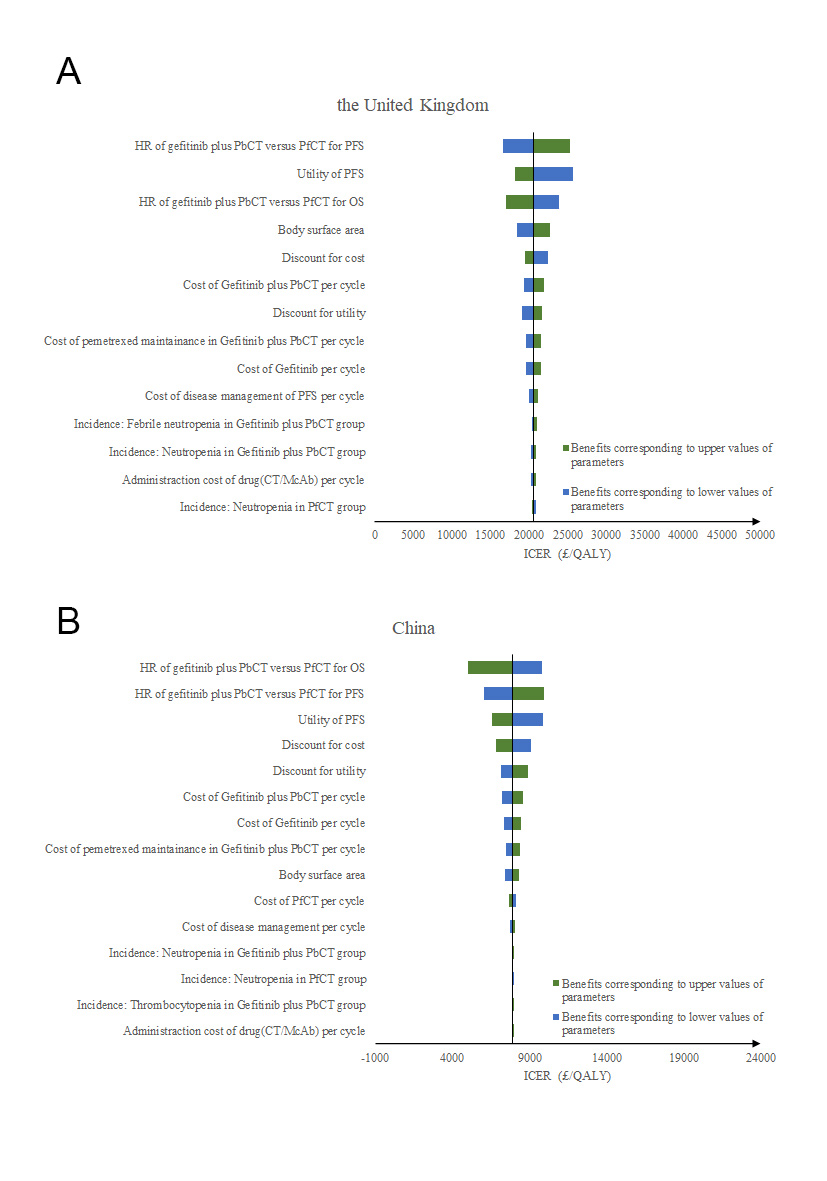


Base-Case ICER: £20609 /QALY

Base-Case ICER: £7895 /QALY

**eFigure 6.** One-way analyses of gefitinib plus pemetrexed based chemotherapy compared with pemetrexed free chemotherapy (A) in the UK setting and (B) in the Chinese setting.

Abbreviation: HR: hazard ratio; PbCT: pemetrexed based chemotherapy; PfCT: pemetrexed free chemotherapy; PFS: progression free survival; OS: overall survival; CT: chemotherapy; McAb: monoclonal antibody

**eTable 1.** The summary of pemetrexed free chemotherapy OS distributions

| Distributions | median OS | mean OS | % at 30 months | % at 60 months | % at 100 months | AIC | BIC |
| --- | --- | --- | --- | --- | --- | --- | --- |
| Weibull | 27.95 | 33.51 | 46.62% | 14.10% | 1.97% | 1238.99 | 1247.74 |
| exponential | 27.69 | 39.95 | 47.19% | 22.27% | 8.18% | 1276.82 | 1281.20 |
| Gompertz | 28.85 | - | 48.27% | 14.30% | 0.73% | 1258.24 | 1266.99 |
| Log-logistic | 27.00 | 49.88 | 45.41% | 19.87% | 9.23% | 1242.26 | 1251.01 |
| Log-normal | 27.42 | 52.32 | 46.84% | 24.54% | 12.75% | 1292.38 | 1301.13 |

**eTable 2.** The summary of pemetrexed free chemotherapy PFS distributions

| Distributions | median PFS | mean PFS | % at 30 months | % at 60 months | % at 100 months | AIC | BIC |
| --- | --- | --- | --- | --- | --- | --- | --- |
| Weibull | 5.59 | 6.36 | 0.01% | 0.00% | 0.00% | 906.89 | 915.22 |
| exponential | 4.87 | 7.03 | 1.40% | 0.02% | 0.02% | 1000.04 | 1004.20 |
| Gompertz | 5.63 | - | 0.00% | 0.00% | 0.00% | 954.06 | 962.38 |
| Log-logistic | 5.30 | 7.53 | 2.01% | 0.43% | 0.43% | 917.42 | 925.74 |
| Log-normal | 5.02 | 7.15 | 1.68% | 0.16% | 0.16% | 945.74 | 954.06 |

**eTable 3.** Pooled grade 3/4 AE incidence rates (%) for 12 first-line treatments for patients with advanced EGFR mutated non-small cell lung cancer

| **Treatments** | **Gefitinib** | **Osimertinib** | **Afatinib** | **Icotinib** | **Dacomitinib** | **Erlotinib** | **Gefitinib+Pemetrexed** | **Erlotinib+Bevazumab** | **Gefitinib+PbCT** | **Afatinib+Cetuximab** | **PbCT** | **PfCT** |
| --- | --- | --- | --- | --- | --- | --- | --- | --- | --- | --- | --- | --- |
| Diarrhea | 2.12 | 2.15 | 11.64 | 0.00 | 8.37 | 1.56 | 0.79 | 3.74 | 3.33 | 15.38 | 0.35 | 0.00 |
| 95% CI | [1.31, 3.25] | [0.79, 4.68] | [9.27, 14.43] | [0, 2.49] | [5.04, 13.07] | [0.67, 3.07] | [0.02, 4.42] | [1.51, 7.71] | [1.34, 6.87] | [7.95, 26.88] | [0.01, 1.93] | [0, 0.64] |
| Fatigue | 0.61 | 0.72 | 1.96 | 0.00 | 0.00 ^a^ | 0.97 | 5.56 | 0.53 | 4.76 | 1.28 | 6.25 | 3.67 |
| 95% CI | [0.22, 1.32] | [0.09, 2.59] | [1.07, 3.29] | [0, 2.49] | [0, 1.63] | [0.32, 2.27] | [2.23, 11.45] | [0.01, 2.98] | [2.28, 8.76] | [0.03, 7.14] | [3.7, 9.88] | [2.27, 5.61] |
| Febrile neutropenia | 0.00 | 0.00^a^ | 0.00 ^a^ | 0.00 ^a^ | 0.00 ^a^ | 0.00 | 0.00 ^a^ | 0.53 | 2.38 | 0.00 ^a^ | 0.00 ^a^ | 0.52 |
| 95% CI | [0, 0.37] | [0, 1.32] | [0, 0.52] | [0, 2.49] | [0, 1.63] | [0, 0.72] | [0, 2.93] | [0.01, 2.98] | [0.77, 5.56] | [0, 4.73] | [0, 1.28] | [0.11, 1.53] |
| Nausea/Vomiting | 1.31 | 0.00 | 2.24 | 0.00 | 2.20 | 0.00 | 1.59 | 0.00 | 2.38 | 2.56 | 4.17 | 6.12 |
| 95% CI | [0.7, 2.25] | [0, 1.32] | [1.28, 3.64] | [0, 2.49] | [0.72, 5.15] | [0, 0.72] | [0.19, 5.74] | [0, 1.97] | [0.77, 5.56] | [0.31, 9.26] | [2.15, 7.28] | [4.26, 8.52] |
| Neutropenia | 0.20 | 0.00 ^a^ | 0.00 | 0.00 | 0.00 ^a^ | 0.39 | 4.76 | 0.53 | 27.14 | 0.00 ^a^ | 13.89 | 45.45 |
| 95% CI | [0.02, 0.73] | [0, 1.32] | [0, 0.52] | [0, 2.49] | [0, 1.63] | [0.05, 1.41] | [1.75, 10.37] | [0.01, 2.98] | [20.56, 35.18] | [0, 4.73] | [9.92, 18.92] | [40.1, 51.34] |
| Rash | 2.63 | 1.08 | 12.48 | 0.00 | 8.81 | 11.50 | 0.00 ^a^ | 22.46 | 5.24 | 39.74 | 0.00 | 0.70 |
| 95% CI | [1.72, 3.85] | [0.22, 3.14] | [10.02, 15.36] | [0, 2.49] | [5.38, 13.61] | [8.76, 14.85] | [0, 2.93] | [16.19, 30.37] | [2.62, 9.37] | [27, 56.42] | [0, 1.28] | [0.19, 1.79] |
| Hypertension | 0.30 | 0.00 ^a^ | 0.00 ^a^ | 0.00 ^a^ | 1.32 | 1.75 | 0.00 ^a^ | 37.97 | 0.95 | 0.00 ^a^ | 0.00 ^a^ | 0.00 ^a^ |
| 95% CI | [0.06, 0.89] | [0, 1.32] | [0, 0.52] | [0, 2.49] | [0.27, 3.86] | [0.8, 3.34] | [0, 2.93] | [29.65, 47.9] | [0.12, 3.44] | [0, 4.73] | [0, 1.28] | [0, 0.64] |
| Leukopenia | 0.10 | 0.00 ^a^ | 0.00 | 0.68 | 0.00 ^a^ | 0.19 | 0.00 | 0.00 ^a^ | 17.14 | 0.00 ^a^ | 5.90 | 15.56 |
| 95% CI | [0, 0.56] | [0, 1.32] | [0, 0.52] | [0.02, 3.76] | [0, 1.63] | [0, 1.09] | [0, 2.93] | [0, 1.97] | [12.01, 23.74] | [0, 4.73] | [3.44, 9.45] | [12.5, 19.16] |
| Anemia | 0.40 | 1.08 | 0.42 | 0.00 | 0.88 | 0.39 | 3.17 | 2.14 | 17.14 | 1.28 | 2.78 | 9.79 |
| 95% CI | [0.11, 1.04] | [0.22, 3.14] | [0.09, 1.23] | [0, 2.49] | [0.11, 3.18] | [0.05, 1.41] | [0.87, 8.13] | [2.2, 9.14] | [12.01, 23.74] | [0.03, 7.14] | [1.2, 5.47] | [7.4, 12.71] |
| Dermatitis | 0.20 | 0.00 | 0.00 ^a^ | 0.00 ^a^ | 15.42 | 0.00 ^a^ | 1.59 | 0.00 ^a^ | 0.00 ^a^ | 0.00 ^a^ | 0.00 ^a^ | 0.00 ^a^ |
| 95% CI | [0.02, 0.73] | [0, 1.32] | [0, 0.52] | [0, 2.49] | [10.74, 21.45] | [0, 0.72] | [0.19, 5.74] | [0, 1.97] | [0, 1.76] | [0, 4.73] | [0, 1.28] | [0, 0.64] |
| ALT/AST rise | 8.70 | 1.08 | 0.84 | 4.73 | 0.88 | 0.97 | 21.43 | 4.81 | 0.00 ^a^ | 2.56 | 0.35 | 1.75 |
| 95% CI | [6.96, 10.74] | [0.22, 3.14] | [0.31, 1.83] | [1.9, 9.75] | [0.11, 3.18] | [0.32, 2.27] | [14.12, 31.19] | [2.2, 9.14] | [0, 1.76] | [0.31, 9.26] | [0.01, 1.93] | [0.84, 3.22] |
| Thrombocytopenia | 0.00 | 0.00 ^a^ | 0.14 | 0.00 ^a^ | 0.00 ^a^ | 0.00 | 0.00 ^a^ | 0.00 ^a^ | 13.81 | 0.00 ^a^ | 0.00 | 11.89 |
| 95% CI | [0, 0.37] | [0, 1.32] | [0, 0.78] | [0, 2.49] | [0, 1.63] | [0, 0.72] | [0, 2.93] | [0, 1.97] | [9.25, 19.84] | [0, 4.73] | [0, 1.28] | [9.23, 15.08] |
| Liver dysfunction | 3.94 | 0.00 ^a^ | 0.00 ^a^ | 0.00 ^a^ | 0.00 | 2.73 | 0.00 ^a^ | 3.21 | 11.90 | 0.00 ^a^ | 0.00 | 0.00 ^a^ |
| 95% CI | [2.8, 5.39] | [0, 1.32] | [0, 0.52] | [0, 2.49] | [0, 1.63] | [1.49, 4.58] | [0, 2.93] | [1.18, 6.99] | [7.7, 17.58] | [0, 4.73] | [0, 1.28] | [0, 0.64] |
| Hair loss | 0.10 | 0.00 | 0.00 | 0.00 ^a^ | 0.44 | 0.00 | 0.00 ^a^ | 0.00 | 0.00 | 0.00 ^a^ | 0.00 ^a^ | 0.35 |
| 95% CI | [0, 0.56] | [0, 1.32] | [0, 0.52] | [0, 2.49] | [0.01, 2.45] | [0, 0.72] | [0, 2.93] | [0, 1.97] | [0, 1.76] | [0, 4.73] | [0, 1.28] | [0.04, 1.26] |

Abbreviation: AE: adverse events; PfCT: pemetrexed-free chemotherapy; PbCT: pemetrexed-based chemotherapy.

^a^the incidence rate of it was assumed to be 0% for not reported in the published articles.

**eTable 4.** Treatment regimens

| **Treatments** | **Regimens** | **Agents** | **Dosages** | **Schedules** |
| --- | --- | --- | --- | --- |
| Gefitinib | Gefitinib | Gefitinib | 250 mg | Once a day |
| Erlotinib | Erlotinib | Erlotinib | 150 mg | Once a day |
| Osimertinib | Osimertinib | Osimertinib | 80 mg | Once a day |
| Afatinib | Afatinib | Afatinib | 40 mg | Once a day |
| Dacomitinib | Dacomitinib | Dacomitinib | 45 mg | Three times a day |
| Icotinib | Icotinib | Icotinib | 125 mg | Three times a day |
| Erlotinib+Bevacizumab | Erlotinib | Erlotinib | 150 mg | Once a day |
|  | Bevacizumab | Bevacizumab | 15 mg/kg | Every 3 weeks |
| Afatinib+Cetuximab | Afatinib+Cetuximab | Afatinib | 40 mg | Once a day |
|  |  | Cetuximab | 500 mg/m^2^ | Once every 2 weeks |
| Gefitinib+PbCT | Gefitinib+PbCT | Gefi­tinib | 250 mg | Once a day |
|  |  | Carboplatin | 400 mg/m^2^ | day 1 of a 21-day cycle (4 cycles) |
|  |  | Pemetrexed | 500 mg/m^2^ | day 1 of a 21-day cycle (4 cycles) |
|  |  | Pemetrexed (maintenance) | 500 mg/m^2^ | Once every 4 weeks (4 cycles) |
| Gefitinib+Pemetrexed | Gefitinib+Pemetrexed | Gefitinib | 250 mg | Once a day |
|  |  | Pemetrexed | 500 mg/m^2^ | Once every 3 weeks (4 cycles) |
| PbCT | Carboplatin+Pemetrexed+Pemetrexed(maintenance) | Carboplatin | 400 mg/m2 | Day 1 of a 21-day cycle (4 cycles) |
|  |  | Pemetrexed | 500 mg/m^2^ | Day 1 of a 21-day cycle (4 cycles) |
|  |  | Pemetrexed (maintenance) | 500 mg/m^2^ | Once every 3 weeks (4 cycles) |
|  | Cisplatin+Pemetrexed+Pemetrexed (maintenance) | Cisplatin | 75 mg/m^2^ | Day 1 of a 21-day cycle (4 cycles) |
|  |  | Pemetrexed | 500 mg/m^2^ | Day 1 of a 21-day cycle (4 cycles) |
|  |  | Pemetrexed (maintenance) | 500 mg/m^2^ | Once every 3 weeks (4 cycles) |
|  | Cisplatin+Pemetrexed | Cisplatin | 75 mg/m^2^ | Day 1 of a 21-day cycle (4 cycles) |
|  |  | Pemetrexed | 500 mg/m^2^ | Day 1 of a 21-day cycle (4 cycles) |
| PfCT | Gemcitabine+Cisplatin | Gemcitabine | 1250 mg/m^2^ | Day 1 and 8 of a 21-day cycle (4 cycles) |
|  |  | Cisplatin | 75 mg/m^2^ | Day 1 of a 21-day cycle (4 cycles) |
|  | Paclitaxel+Carboplatin | Paclitaxel | 200 mg/m^2^ | Day 1 of a 21-day cycle (4 cycles) |
|  |  | Carboplatin | 400 mg/m^2^ | Day 1 of a 21-day cycle (4 cycles) |
|  | Gemcitabine+Cisplatin | Gemcitabine | 1000 mg/m^2^ | Day 1 and 8 of a 21-day cycle (4 cycles) |
|  |  | Cisplatin | 75 mg/m^2^ | Day 1 of a 21-day cycle (4 cycles) |
|  | Docetaxel+Cisplatin | Cisplatin | 80 mg/m^2^ | Day 1 of a 21-day cycle (4 cycles) |
|  |  | Docetaxel | 60 mg/m^2^ | Day 1 of a 21-day cycle (4 cycles) |
|  | Gemcitabine/Docetaxel+Cisplatin | Gemcitabine | 1250 mg/m^2^ | Day 1 and 8 of a 21-day cycle (4 cycles) |
|  |  | Cisplatin | 75 mg/m^2^ | Day 1 of a 21-day cycle (4 cycles) |
|  |  | Docetaxel | 75 mg/m^2^ | Day 1 of a 21-day cycle (4 cycles) |
|  | Gemcitabine+Carboplatin | Gemcitabine | 1250 mg/m^2^ | Day 1 and 8 of a 21-day cycle (4 cycles) |
|  |  | Carboplatin | 400 mg/m^2^ | Day 1 of a 21-day cycle (4 cycles) |
| Subsequent therapies | Cisplatin+Pemetrexed | Cisplatin | 75 mg/m^2^ | Day 1 of a 21-day cycle (4 cycles) |
|  |  | Pemetrexed | 500 mg/m^2^ | Day 1 of a 21-day cycle (4 cycles) |
|  | Docetaxel | Docetaxel | 75 mg/m^2^ | Day 1 of a 21-day cycle (4 cycles) |

Abbreviation: PbCT: pemetrexed based chemotherapy; PfCT: pemetrexed free chemotherapy.

**eTable 5.** Estimated costs and QALYs caused by specific SAEs per patient in the UK

| **Treatments** | **SAE** | **Incidence** | **Unit price** | **SAE costs** | **Total SAE costs** | **Disutility per event** | **SAE Disutility** | **Total Disutility^a^** |
| --- | --- | --- | --- | --- | --- | --- | --- | --- |
| Gefitinib | Diarrhea | 2.12% | 1241 | 26.36 | 76.75 | 0.320 | 0.0016 | 0.0042 |
|  | Fatigue | 0.61% | 2638 | 16.01 |  | 0.410 | 0.0006 |  |
|  | Febrile neutropenia | 0.00% | 11687 | 0.00 |  | 0.500 | 0.0000 |  |
|  | Nausea/Vomiting | 1.31% | 1241 | 16.32 |  | 0.250 | 0.0008 |  |
|  | Neutropenia | 0.20% | 2048 | 4.14 |  | 0.460 | 0.0002 |  |
|  | Rash | 2.63% | 130 | 3.41 |  | 0.150 | 0.0009 |  |
|  | Hypertension | 0.30% | 2212 | 6.71 |  | 0.050 | 0.0000 |  |
|  | Leukopenia | 0.10% | 322 | 0.33 |  | 0.200 | 0.0000 |  |
|  | Anemia | 0.40% | 796 | 3.22 |  | 0.074 | 0.0001 |  |
|  | Dermatitis | 0.20% | 130 | 0.26 |  | 0.032 | 0.0000 |  |
|  | Thrombocytopenia | 0.00% | 327 | 0.00 |  | 0.200 | 0.0000 |  |
|  | Hair loss | 0.10% | 0 | 0.00 |  | 0.130 | 0.0000 |  |
| Osimertinib | Diarrhea | 2.15% | 1241 | 26.69 | 55.56 | 0.320 | 0.0016 | 0.0028 |
|  | Fatigue | 0.72% | 2638 | 18.91 |  | 0.410 | 0.0007 |  |
|  | Febrile neutropenia | 0.00% | 11687 | 0.00 |  | 0.500 | 0.0000 |  |
|  | Nausea/Vomiting | 0.00% | 1241 | 0.00 |  | 0.250 | 0.0000 |  |
|  | Neutropenia | 0.00% | 2048 | 0.00 |  | 0.460 | 0.0000 |  |
|  | Rash | 1.08% | 130 | 1.39 |  | 0.150 | 0.0004 |  |
|  | Hypertension | 0.00% | 2212 | 0.00 |  | 0.050 | 0.0000 |  |
|  | Leukopenia | 0.00% | 322 | 0.00 |  | 0.200 | 0.0000 |  |
|  | Anemia | 1.08% | 796 | 8.56 |  | 0.074 | 0.0002 |  |
|  | Dermatitis | 0.00% | 130 | 0.00 |  | 0.032 | 0.0000 |  |
|  | Thrombocytopenia | 0.00% | 327 | 0.00 |  | 0.200 | 0.0000 |  |
|  | Hair loss | 0.00% | 0 | 0.00 |  | 0.130 | 0.0000 |  |
| Afatinib | Diarrhea | 11.64% | 1241 | 144.50 | 244.15 | 0.320 | 0.0086 | 0.0162 |
|  | Fatigue | 1.96% | 2638 | 51.81 |  | 0.410 | 0.0019 |  |
|  | Febrile neutropenia | 0.00% | 11687 | 0.00 |  | 0.500 | 0.0000 |  |
|  | Nausea/Vomiting | 2.24% | 1241 | 27.86 |  | 0.250 | 0.0013 |  |
|  | Neutropenia | 0.00% | 2048 | 0.00 |  | 0.460 | 0.0000 |  |
|  | Rash | 12.48% | 130 | 16.19 |  | 0.150 | 0.0043 |  |
|  | Hypertension | 0.00% | 2212 | 0.00 |  | 0.050 | 0.0000 |  |
|  | Leukopenia | 0.00% | 322 | 0.00 |  | 0.200 | 0.0000 |  |
|  | Anemia | 0.42% | 796 | 3.35 |  | 0.074 | 0.0001 |  |
|  | Dermatitis | 0.00% | 130 | 0.00 |  | 0.032 | 0.0000 |  |
|  | Thrombocytopenia | 0.14% | 327 | 0.46 |  | 0.200 | 0.0001 |  |
|  | Hair loss | 0.00% | 0 | 0.00 |  | 0.130 | 0.0000 |  |
| Dacomitinib | Diarrhea | 8.37% | 1241 | 103.90 | 198.90 | 0.320 | 0.0062 | 0.0121 |
|  | Fatigue | 0.00% | 2638 | 0.00 |  | 0.410 | 0.0000 |  |
|  | Febrile neutropenia | 0.00% | 11687 | 0.00 |  | 0.500 | 0.0000 |  |
|  | Nausea/Vomiting | 2.20% | 1241 | 27.34 |  | 0.250 | 0.0013 |  |
|  | Neutropenia | 0.00% | 2048 | 0.00 |  | 0.460 | 0.0000 |  |
|  | Rash | 8.81% | 130 | 11.43 |  | 0.150 | 0.0030 |  |
|  | Hypertension | 1.32% | 2212 | 29.24 |  | 0.050 | 0.0002 |  |
|  | Leukopenia | 0.00% | 322 | 0.00 |  | 0.200 | 0.0000 |  |
|  | Anemia | 0.88% | 796 | 7.01 |  | 0.074 | 0.0001 |  |
|  | Dermatitis | 15.42% | 130 | 19.99 |  | 0.032 | 0.0012 |  |
|  | Thrombocytopenia | 0.00% | 327 | 0.00 |  | 0.200 | 0.0000 |  |
|  | Hair loss | 0.44% | 0 | 0.00 |  | 0.130 | 0.0001 |  |
| Erlotinib | Diarrhea | 1.56% | 1241 | 19.36 | 110.51 | 0.320 | 0.0012 | 0.0068 |
|  | Fatigue | 0.97% | 2638 | 25.72 |  | 0.410 | 0.0009 |  |
|  | Febrile neutropenia | 0.00% | 11687 | 0.00 |  | 0.500 | 0.0000 |  |
|  | Nausea/Vomiting | 0.00% | 1241 | 0.00 |  | 0.250 | 0.0000 |  |
|  | Neutropenia | 0.39% | 2048 | 7.98 |  | 0.460 | 0.0004 |  |
|  | Rash | 11.50% | 130 | 14.91 |  | 0.150 | 0.0040 |  |
|  | Hypertension | 1.75% | 2212 | 38.81 |  | 0.050 | 0.0002 |  |
|  | Leukopenia | 0.19% | 322 | 0.63 |  | 0.200 | 0.0001 |  |
|  | Anemia | 0.39% | 796 | 3.10 |  | 0.074 | 0.0001 |  |
|  | Dermatitis | 0.00% | 130 | 0.00 |  | 0.032 | 0.0000 |  |
|  | Thrombocytopenia | 0.00% | 327 | 0.00 |  | 0.200 | 0.0000 |  |
|  | Hair loss | 0.00% | 0 | 0.00 |  | 0.130 | 0.0000 |  |
| Afatinib+Cetuximab | Diarrhea | 15.38% | 1241 | 190.97 | 318.36 | 0.320 | 0.0114 | 0.0280 |
|  | Fatigue | 1.28% | 2638 | 33.83 |  | 0.410 | 0.0012 |  |
|  | Febrile neutropenia | 0.00% | 11687 | 0.00 |  | 0.500 | 0.0000 |  |
|  | Nausea/Vomiting | 2.56% | 1241 | 31.83 |  | 0.250 | 0.0015 |  |
|  | Neutropenia | 0.00% | 2048 | 0.00 |  | 0.460 | 0.0000 |  |
|  | Rash | 39.74% | 130 | 51.54 |  | 0.150 | 0.0138 |  |
|  | Hypertension | 0.00% | 2212 | 0.00 |  | 0.050 | 0.0000 |  |
|  | Leukopenia | 0.00% | 322 | 0.00 |  | 0.200 | 0.0000 |  |
|  | Anemia | 1.28% | 796 | 10.20 |  | 0.074 | 0.0002 |  |
|  | Dermatitis | 0.00% | 130 | 0.00 |  | 0.032 | 0.0000 |  |
|  | Thrombocytopenia | 0.00% | 327 | 0.00 |  | 0.200 | 0.0000 |  |
|  | Hair loss | 0.00% | 0 | 0.00 |  | 0.130 | 0.0000 |  |
| Erlotinib+Bevacizumab | Diarrhea | 3.74% | 1241 | 46.47 | 1020.11 | 0.320 | 0.0028 | 0.0170 |
|  | Fatigue | 0.53% | 2638 | 14.11 |  | 0.410 | 0.0005 |  |
|  | Febrile neutropenia | 0.53% | 11687 | 62.50 |  | 0.500 | 0.0006 |  |
|  | Nausea/Vomiting | 0.00% | 1241 | 0.00 |  | 0.250 | 0.0000 |  |
|  | Neutropenia | 0.53% | 2048 | 10.95 |  | 0.460 | 0.0006 |  |
|  | Rash | 22.46% | 130 | 29.12 |  | 0.150 | 0.0078 |  |
|  | Hypertension | 37.97% | 2212 | 839.94 |  | 0.050 | 0.0044 |  |
|  | Leukopenia | 0.00% | 322 | 0.00 |  | 0.200 | 0.0000 |  |
|  | Anemia | 2.14% | 796 | 17.02 |  | 0.074 | 0.0004 |  |
|  | Dermatitis | 0.00% | 130 | 0.00 |  | 0.032 | 0.0000 |  |
|  | Thrombocytopenia | 0.00% | 327 | 0.00 |  | 0.200 | 0.0000 |  |
|  | Hair loss | 0.00% | 0 | 0.00 |  | 0.130 | 0.0000 |  |
| Gefitinib+Pemetrexed | Diarrhea | 0.79% | 1241 | 9.85 | 300.95 | 0.320 | 0.0006 | 0.0125 |
|  | Fatigue | 5.56% | 2638 | 146.58 |  | 0.410 | 0.0053 |  |
|  | Febrile neutropenia | 0.00% | 11687 | 0.00 |  | 0.500 | 0.0000 |  |
|  | Nausea/Vomiting | 1.59% | 1241 | 19.70 |  | 0.250 | 0.0009 |  |
|  | Neutropenia | 4.76% | 2048 | 97.50 |  | 0.460 | 0.0051 |  |
|  | Rash | 0.00% | 130 | 0.00 |  | 0.150 | 0.0000 |  |
|  | Hypertension | 0.00% | 2212 | 0.00 |  | 0.050 | 0.0000 |  |
|  | Leukopenia | 0.00% | 322 | 0.00 |  | 0.200 | 0.0000 |  |
|  | Anemia | 3.17% | 796 | 25.26 |  | 0.074 | 0.0005 |  |
|  | Dermatitis | 1.59% | 130 | 2.06 |  | 0.032 | 0.0001 |  |
|  | Thrombocytopenia | 0.00% | 327 | 0.00 |  | 0.200 | 0.0000 |  |
|  | Hair loss | 0.00% | 0 | 0.00 |  | 0.130 | 0.0000 |  |
| Gefitinib+PbCT | Diarrhea | 3.33% | 1241 | 41.38 | 1295.19 | 0.320 | 0.0025 | 0.0590 |
|  | Fatigue | 4.76% | 2638 | 125.64 |  | 0.410 | 0.0045 |  |
|  | Febrile neutropenia | 2.38% | 11687 | 278.27 |  | 0.500 | 0.0027 |  |
|  | Nausea/Vomiting | 2.38% | 1241 | 29.55 |  | 0.250 | 0.0014 |  |
|  | Neutropenia | 27.14% | 2048 | 555.75 |  | 0.460 | 0.0288 |  |
|  | Rash | 5.24% | 130 | 6.79 |  | 0.150 | 0.0018 |  |
|  | Hypertension | 0.95% | 2212 | 21.07 |  | 0.050 | 0.0001 |  |
|  | Leukopenia | 17.14% | 322 | 55.18 |  | 0.200 | 0.0079 |  |
|  | Anemia | 17.14% | 796 | 136.41 |  | 0.074 | 0.0029 |  |
|  | Dermatitis | 0.00% | 130 | 0.00 |  | 0.032 | 0.0000 |  |
|  | Thrombocytopenia | 13.81% | 327 | 45.15 |  | 0.200 | 0.0064 |  |
|  | Hair loss | 0.00% | 0 | 0.00 |  | 0.130 | 0.0000 |  |
| PbCT | Diarrhea | 0.35% | 1241 | 4.31 | 546.41 | 0.320 | 0.0003 | 0.0265 |
|  | Fatigue | 6.25% | 2638 | 164.90 |  | 0.410 | 0.0059 |  |
|  | Febrile neutropenia | 0.00% | 11687 | 0.00 |  | 0.500 | 0.0000 |  |
|  | Nausea/Vomiting | 4.17% | 1241 | 51.72 |  | 0.250 | 0.0024 |  |
|  | Neutropenia | 13.89% | 2048 | 284.38 |  | 0.460 | 0.0147 |  |
|  | Rash | 0.00% | 130 | 0.00 |  | 0.150 | 0.0000 |  |
|  | Hypertension | 0.00% | 2212 | 0.00 |  | 0.050 | 0.0000 |  |
|  | Leukopenia | 5.90% | 322 | 19.00 |  | 0.200 | 0.0027 |  |
|  | Anemia | 2.78% | 796 | 22.10 |  | 0.074 | 0.0005 |  |
|  | Dermatitis | 0.00% | 130 | 0.00 |  | 0.032 | 0.0000 |  |
|  | Thrombocytopenia | 0.00% | 327 | 0.00 |  | 0.200 | 0.0000 |  |
|  | Hair loss | 0.00% | 0 | 0.00 |  | 0.130 | 0.0000 |  |
| PfCT | Diarrhea | 0.00% | 1241 | 0.00 | 1332.56 | 0.320 | 0.0000 | 0.0705 |
|  | Fatigue | 3.67% | 2638 | 96.86 |  | 0.410 | 0.0035 |  |
|  | Febrile neutropenia | 0.52% | 11687 | 61.30 |  | 0.500 | 0.0006 |  |
|  | Nausea/Vomiting | 6.12% | 1241 | 75.95 |  | 0.250 | 0.0035 |  |
|  | Neutropenia | 45.45% | 2048 | 930.68 |  | 0.460 | 0.0483 |  |
|  | Rash | 0.70% | 130 | 0.91 |  | 0.150 | 0.0002 |  |
|  | Hypertension | 0.00% | 2212 | 0.00 |  | 0.050 | 0.0000 |  |
|  | Leukopenia | 15.56% | 322 | 50.08 |  | 0.200 | 0.0072 |  |
|  | Anemia | 9.79% | 796 | 77.90 |  | 0.074 | 0.0017 |  |
|  | Dermatitis | 0.00% | 130 | 0.00 |  | 0.032 | 0.0000 |  |
|  | Thrombocytopenia | 11.89% | 327 | 38.87 |  | 0.200 | 0.0055 |  |
|  | Hair loss | 0.35% | 0 | 0.00 |  | 0.130 | 0.0001 |  |

Abbreviation: SAE: serious adverse event; PbCT: pemetrexed based chemotherapy; PfCT pemetrexed free chemotherapy.

^a^each serious adverse event was assumed to lasting 3 months through the treatment, which was the same length as the chemotherapies dosing schedule.

**eTable 6.** Estimated costs (£) and QALYs caused by specific SAEs per patient in China

| **Treatments** | **SAE** | **Incidence** | **Unit price** | **SAE costs** | **Total SAE costs** | **Disutility per event** | **SAE Disutility** | **Total Disutility^a^** |
| --- | --- | --- | --- | --- | --- | --- | --- | --- |
| Gefitinib | Diarrhea | 2.12% | 4 | 0.09 | 4.29 | 0.070 | 0.0003 | 0.0017 |
|  | Fatigue | 0.61% | 99 | 0.60 |  | 0.070 | 0.0001 |  |
|  | Febrile neutropenia | 0.00% | 869 | 0.00 |  | 0.420 | 0.0000 |  |
|  | Nausea/Vomiting | 1.31% | 54 | 0.71 |  | 0.120 | 0.0004 |  |
|  | Neutropenia | 0.20% | 396 | 0.80 |  | 0.200 | 0.0001 |  |
|  | Rash | 2.63% | 4 | 0.12 |  | 0.100 | 0.0006 |  |
|  | Hypertension | 0.30% | 10 | 0.03 |  | 0.040 | 0.0000 |  |
|  | Leukopenia | 0.10% | 82 | 0.08 |  | 0.200 | 0.0000 |  |
|  | Anemia | 0.40% | 456 | 1.84 |  | 0.090 | 0.0001 |  |
|  | Dermatitis | 0.20% | 4 | 0.01 |  | 0.032 | 0.0000 |  |
|  | Thrombocytopenia | 0.00% | 416 | 0.00 |  | 0.200 | 0.0000 |  |
|  | Hair loss | 0.10% | 0 | 0.00 |  | 0.060 | 0.0000 |  |
| Osimertinib | Diarrhea | 2.15% | 4 | 0.10 | 5.75 | 0.070 | 0.0003 | 0.0009 |
|  | Fatigue | 0.72% | 99 | 0.71 |  | 0.070 | 0.0001 |  |
|  | Febrile neutropenia | 0.00% | 869 | 0.00 |  | 0.420 | 0.0000 |  |
|  | Nausea/Vomiting | 0.00% | 54 | 0.00 |  | 0.120 | 0.0000 |  |
|  | Neutropenia | 0.00% | 396 | 0.00 |  | 0.200 | 0.0000 |  |
|  | Rash | 1.08% | 4 | 0.05 |  | 0.100 | 0.0002 |  |
|  | Hypertension | 0.00% | 10 | 0.00 |  | 0.040 | 0.0000 |  |
|  | Leukopenia | 0.00% | 82 | 0.00 |  | 0.200 | 0.0000 |  |
|  | Anemia | 1.08% | 456 | 4.90 |  | 0.090 | 0.0002 |  |
|  | Dermatitis | 0.00% | 4 | 0.00 |  | 0.032 | 0.0000 |  |
|  | Thrombocytopenia | 0.00% | 416 | 0.00 |  | 0.200 | 0.0000 |  |
|  | Hair loss | 0.00% | 0 | 0.00 |  | 0.060 | 0.0000 |  |
| Afatinib | Diarrhea | 11.64% | 4 | 0.52 | 6.72 | 0.070 | 0.0019 | 0.0059 |
|  | Fatigue | 1.96% | 99 | 1.94 |  | 0.070 | 0.0003 |  |
|  | Febrile neutropenia | 0.00% | 869 | 0.00 |  | 0.420 | 0.0000 |  |
|  | Nausea/Vomiting | 2.24% | 54 | 1.21 |  | 0.120 | 0.0006 |  |
|  | Neutropenia | 0.00% | 396 | 0.00 |  | 0.200 | 0.0000 |  |
|  | Rash | 12.48% | 4 | 0.56 |  | 0.100 | 0.0029 |  |
|  | Hypertension | 0.00% | 10 | 0.00 |  | 0.040 | 0.0000 |  |
|  | Leukopenia | 0.00% | 82 | 0.00 |  | 0.200 | 0.0000 |  |
|  | Anemia | 0.42% | 456 | 1.92 |  | 0.090 | 0.0001 |  |
|  | Dermatitis | 0.00% | 4 | 0.00 |  | 0.032 | 0.0000 |  |
|  | Thrombocytopenia | 0.14% | 416 | 0.58 |  | 0.200 | 0.0001 |  |
|  | Hair loss | 0.00% | 0 | 0.00 |  | 0.060 | 0.0000 |  |
| Icotinib | Diarrhea | 0.00% | 4 | 0.00 | 0.56 | 0.070 | 0.0000 | 0.0003 |
|  | Fatigue | 0.00% | 99 | 0.00 |  | 0.070 | 0.0000 |  |
|  | Febrile neutropenia | 0.00% | 869 | 0.00 |  | 0.420 | 0.0000 |  |
|  | Nausea/Vomiting | 0.00% | 54 | 0.00 |  | 0.120 | 0.0000 |  |
|  | Neutropenia | 0.00% | 396 | 0.00 |  | 0.200 | 0.0000 |  |
|  | Rash | 0.00% | 4 | 0.00 |  | 0.100 | 0.0000 |  |
|  | Hypertension | 0.00% | 10 | 0.00 |  | 0.040 | 0.0000 |  |
|  | Leukopenia | 0.68% | 82 | 0.56 |  | 0.200 | 0.0003 |  |
|  | Anemia | 0.00% | 456 | 0.00 |  | 0.090 | 0.0000 |  |
|  | Dermatitis | 0.00% | 4 | 0.00 |  | 0.032 | 0.0000 |  |
|  | Thrombocytopenia | 0.00% | 416 | 0.00 |  | 0.200 | 0.0000 |  |
|  | Hair loss | 0.00% | 0 | 0.00 |  | 0.060 | 0.0000 |  |
| Dacomitinib | Diarrhea | 8.37% | 4 | 0.37 | 6.79 | 0.070 | 0.0014 | 0.0055 |
|  | Fatigue | 0.00% | 99 | 0.00 |  | 0.070 | 0.0000 |  |
|  | Febrile neutropenia | 0.00% | 869 | 0.00 |  | 0.420 | 0.0000 |  |
|  | Nausea/Vomiting | 2.20% | 54 | 1.19 |  | 0.120 | 0.0006 |  |
|  | Neutropenia | 0.00% | 396 | 0.00 |  | 0.200 | 0.0000 |  |
|  | Rash | 8.81% | 4 | 0.39 |  | 0.100 | 0.0020 |  |
|  | Hypertension | 1.32% | 10 | 0.13 |  | 0.040 | 0.0001 |  |
|  | Leukopenia | 0.00% | 82 | 0.00 |  | 0.200 | 0.0000 |  |
|  | Anemia | 0.88% | 456 | 4.02 |  | 0.090 | 0.0002 |  |
|  | Dermatitis | 15.42% | 4 | 0.69 |  | 0.032 | 0.0012 |  |
|  | Thrombocytopenia | 0.00% | 416 | 0.00 |  | 0.200 | 0.0000 |  |
|  | Hair loss | 0.44% | 0 | 0.00 |  | 0.060 | 0.0001 |  |
| Erlotinib | Diarrhea | 1.56% | 4 | 0.07 | 5.20 | 0.070 | 0.0003 | 0.0036 |
|  | Fatigue | 0.97% | 99 | 0.96 |  | 0.070 | 0.0002 |  |
|  | Febrile neutropenia | 0.00% | 869 | 0.00 |  | 0.420 | 0.0000 |  |
|  | Nausea/Vomiting | 0.00% | 54 | 0.00 |  | 0.120 | 0.0000 |  |
|  | Neutropenia | 0.39% | 396 | 1.54 |  | 0.200 | 0.0002 |  |
|  | Rash | 11.50% | 4 | 0.51 |  | 0.100 | 0.0027 |  |
|  | Hypertension | 1.75% | 10 | 0.18 |  | 0.040 | 0.0002 |  |
|  | Leukopenia | 0.19% | 82 | 0.16 |  | 0.200 | 0.0001 |  |
|  | Anemia | 0.39% | 456 | 1.78 |  | 0.090 | 0.0001 |  |
|  | Dermatitis | 0.00% | 4 | 0.00 |  | 0.032 | 0.0000 |  |
|  | Thrombocytopenia | 0.00% | 416 | 0.00 |  | 0.200 | 0.0000 |  |
|  | Hair loss | 0.00% | 0 | 0.00 |  | 0.060 | 0.0000 |  |
| Afatinib+Cetuximab | Diarrhea | 15.38% | 4 | 0.69 | 10.95 | 0.070 | 0.0025 | 0.0128 |
|  | Fatigue | 1.28% | 99 | 1.27 |  | 0.070 | 0.0002 |  |
|  | Febrile neutropenia | 0.00% | 869 | 0.00 |  | 0.420 | 0.0000 |  |
|  | Nausea/Vomiting | 2.56% | 54 | 1.38 |  | 0.120 | 0.0007 |  |
|  | Neutropenia | 0.00% | 396 | 0.00 |  | 0.200 | 0.0000 |  |
|  | Rash | 39.74% | 4 | 1.77 |  | 0.100 | 0.0092 |  |
|  | Hypertension | 0.00% | 10 | 0.00 |  | 0.040 | 0.0000 |  |
|  | Leukopenia | 0.00% | 82 | 0.00 |  | 0.200 | 0.0000 |  |
|  | Anemia | 1.28% | 456 | 5.85 |  | 0.090 | 0.0003 |  |
|  | Dermatitis | 0.00% | 4 | 0.00 |  | 0.032 | 0.0000 |  |
|  | Thrombocytopenia | 0.00% | 416 | 0.00 |  | 0.200 | 0.0000 |  |
|  | Hair loss | 0.00% | 0 | 0.00 |  | 0.060 | 0.0000 |  |
| Erlotinib+Bevacizumab | Diarrhea | 3.74% | 4 | 0.17 | 22.06 | 0.070 | 0.0006 | 0.0106 |
|  | Fatigue | 0.53% | 99 | 0.53 |  | 0.070 | 0.0001 |  |
|  | Febrile neutropenia | 0.53% | 869 | 4.65 |  | 0.420 | 0.0005 |  |
|  | Nausea/Vomiting | 0.00% | 54 | 0.00 |  | 0.120 | 0.0000 |  |
|  | Neutropenia | 0.53% | 396 | 2.12 |  | 0.200 | 0.0002 |  |
|  | Rash | 22.46% | 4 | 1.00 |  | 0.100 | 0.0052 |  |
|  | Hypertension | 37.97% | 10 | 3.85 |  | 0.040 | 0.0035 |  |
|  | Leukopenia | 0.00% | 82 | 0.00 |  | 0.200 | 0.0000 |  |
|  | Anemia | 2.14% | 456 | 9.75 |  | 0.090 | 0.0004 |  |
|  | Dermatitis | 0.00% | 4 | 0.00 |  | 0.032 | 0.0000 |  |
|  | Thrombocytopenia | 0.00% | 416 | 0.00 |  | 0.200 | 0.0000 |  |
|  | Hair loss | 0.00% | 0 | 0.00 |  | 0.060 | 0.0000 |  |
| Gefitinib+Pemetrexed | Diarrhea | 0.79% | 4 | 0.04 | 39.77 | 0.070 | 0.0001 | 0.0044 |
|  | Fatigue | 5.56% | 99 | 5.49 |  | 0.070 | 0.0009 |  |
|  | Febrile neutropenia | 0.00% | 869 | 0.00 |  | 0.420 | 0.0000 |  |
|  | Nausea/Vomiting | 1.59% | 54 | 0.85 |  | 0.120 | 0.0004 |  |
|  | Neutropenia | 4.76% | 396 | 18.85 |  | 0.200 | 0.0022 |  |
|  | Rash | 0.00% | 4 | 0.00 |  | 0.100 | 0.0000 |  |
|  | Hypertension | 0.00% | 10 | 0.00 |  | 0.040 | 0.0000 |  |
|  | Leukopenia | 0.00% | 82 | 0.00 |  | 0.200 | 0.0000 |  |
|  | Anemia | 3.17% | 456 | 14.47 |  | 0.090 | 0.0007 |  |
|  | Dermatitis | 1.59% | 4 | 0.07 |  | 0.032 | 0.0001 |  |
|  | Thrombocytopenia | 0.00% | 416 | 0.00 |  | 0.200 | 0.0000 |  |
|  | Hair loss | 0.00% | 0 | 0.00 |  | 0.060 | 0.0000 |  |
| Gefitinib+PbCT | Diarrhea | 0.00% | 4 | 0.00 | 284.30 | 0.070 | 0.0000 | 0.0359 |
|  | Fatigue | 0.00% | 99 | 0.00 |  | 0.070 | 0.0000 |  |
|  | Febrile neutropenia | 0.00% | 869 | 0.00 |  | 0.420 | 0.0000 |  |
|  | Nausea/Vomiting | 1.08% | 54 | 0.58 |  | 0.120 | 0.0003 |  |
|  | Neutropenia | 0.00% | 396 | 0.00 |  | 0.200 | 0.0000 |  |
|  | Rash | 0.00% | 4 | 0.00 |  | 0.100 | 0.0000 |  |
|  | Hypertension | 1.08% | 10 | 0.11 |  | 0.040 | 0.0001 |  |
|  | Leukopenia | 0.00% | 82 | 0.00 |  | 0.200 | 0.0000 |  |
|  | Anemia | 0.00% | 456 | 0.00 |  | 0.090 | 0.0000 |  |
|  | Dermatitis | 0.00% | 4 | 0.00 |  | 0.032 | 0.0000 |  |
|  | Thrombocytopenia | 2.12% | 416 | 8.83 |  | 0.200 | 0.0010 |  |
|  | Hair loss | 0.61% | 0 | 0.00 |  | 0.060 | 0.0001 |  |
| PbCT | Diarrhea | 0.35% | 4 | 0.02 | 80.93 | 0.070 | 0.0001 | 0.0119 |
|  | Fatigue | 6.25% | 99 | 6.17 |  | 0.070 | 0.0010 |  |
|  | Febrile neutropenia | 0.00% | 869 | 0.00 |  | 0.420 | 0.0000 |  |
|  | Nausea/Vomiting | 4.17% | 54 | 2.24 |  | 0.120 | 0.0012 |  |
|  | Neutropenia | 13.89% | 396 | 54.97 |  | 0.200 | 0.0064 |  |
|  | Rash | 0.00% | 4 | 0.00 |  | 0.100 | 0.0000 |  |
|  | Hypertension | 0.00% | 10 | 0.00 |  | 0.040 | 0.0000 |  |
|  | Leukopenia | 5.90% | 82 | 4.87 |  | 0.200 | 0.0027 |  |
|  | Anemia | 2.78% | 456 | 12.67 |  | 0.090 | 0.0006 |  |
|  | Dermatitis | 0.00% | 4 | 0.00 |  | 0.032 | 0.0000 |  |
|  | Thrombocytopenia | 0.00% | 416 | 0.00 |  | 0.200 | 0.0000 |  |
|  | Hair loss | 0.00% | 0 | 0.00 |  | 0.060 | 0.0000 |  |
| PfCT | Diarrhea | 0.00% | 4 | 0.00 | 298.31 | 0.070 | 0.0000 | 0.0387 |
|  | Fatigue | 3.67% | 99 | 3.63 |  | 0.070 | 0.0006 |  |
|  | Febrile neutropenia | 0.52% | 869 | 4.56 |  | 0.420 | 0.0005 |  |
|  | Nausea/Vomiting | 6.12% | 54 | 3.29 |  | 0.120 | 0.0017 |  |
|  | Neutropenia | 45.45% | 396 | 179.91 |  | 0.200 | 0.0210 |  |
|  | Rash | 0.70% | 4 | 0.03 |  | 0.100 | 0.0002 |  |
|  | Hypertension | 0.00% | 10 | 0.00 |  | 0.040 | 0.0000 |  |
|  | Leukopenia | 15.56% | 82 | 12.82 |  | 0.200 | 0.0072 |  |
|  | Anemia | 9.79% | 456 | 44.64 |  | 0.090 | 0.0020 |  |
|  | Dermatitis | 0.00% | 4 | 0.00 |  | 0.032 | 0.0000 |  |
|  | Thrombocytopenia | 11.89% | 416 | 49.44 |  | 0.200 | 0.0055 |  |
|  | Hair loss | 0.35% | 0 | 0.00 |  | 0.060 | 0.0000 |  |

Abbreviation: SAE: serious adverse event; PbCT: pemetrexed based chemotherapy; PfCT pemetrexed free chemotherapy.

^a^each serious adverse event was assumed to lasting 3 months through the treatment, which was the same length as the chemotherapies dosing schedule.

**eTable 7.** Scenario 1: summary of cost-effectiveness results in the UK (updated overall survival data for osimertinib).

| **Treatments** | **Costs (£)** | **QALYs** | **LYs** | **ICER^a^**  **(£/QALY)** | **sequential ICER^b^ (£/QALY)** |
| --- | --- | --- | --- | --- | --- |
| Gefitinib | 24529 | 1.130 | 2.571 | -1217 | Dominant |
| Osimertinib | 142163 | 1.818 | 3.821 | 109060 | 745939 |
| Afatinib | 51865 | 1.255 | 2.899 | 52570 | Dominated |
| Dacomitinib | 155510 | 1.475 | 3.080 | 178439 | Dominated |
| Erlotinib | 27237 | 1.177 | 2.554 | 5169 | Extended dominated |
| Afatinib+Cetuximab | 92935 | 1.113 | 2.535 | 184107 | Dominated |
| Erlotinib+Bevacizumab | 106486 | 1.541 | 2.950 | 102246 | Dominated |
| Gefitinib+Pemetrexed | 33221 | 1.401 | 3.080 | 12513 | 32074 |
| Gefitinib+PbCT | 44445 | 1.687 | 3.632 | 20609 | 39245 |
| PbCT | 31595 | 0.861 | 2.426 | 56160 | Dominated |
| PfCT | 24999 | 0.744 | 2.607 | Reference | Dominated |

Abbreviation: PbCT: pemetrexed based chemotherapy; PfCT: pemetrexed free chemotherapy; LY: life year; QALY: quality-adjusted life year; ICER: incremental cost-effectiveness ratio.

^a^compared with PfCT; ^b^sequential ICER was compared with the next best non-dominated option.

**eTable 8.** Scenario 1: summary of cost-effectiveness results in China (updated overall survival data for osimertinib).

| **Treatments** | **Costs (£)** | **QALYs** | **LYs** | **ICER^a^**  **(£/QALY)** | **sequential ICER^b^ (£/QALY)** |
| --- | --- | --- | --- | --- | --- |
| Gefitinib | 12961 | 1.312 | 2.572 | -7266 | Dominant |
| Osimertinib | 27246 | 2.044 | 3.822 | 12690 | 45424 |
| Afatinib | 21478 | 1.469 | 2.900 | 16418 | Dominated |
| Icotinib | 18308 | 1.254 | 2.482 | 18310 | Dominated |
| Dacomitinib | 22517 | 1.656 | 3.081 | 13009 | Dominated |
| Erlotinib | 19270 | 1.340 | 2.554 | 16118 | Dominated |
| Afatinib+Cetuximab | 52380 | 1.297 | 2.536 | 159297 | Dominated |
| Erlotinib+Bevacizumab | 58504 | 1.673 | 2.951 | 71527 | Dominated |
| Gefitinib+Pemetrexed | 16873 | 1.606 | 3.081 | 3842 | 13290 |
| Gefitinib+PbCT | 21545 | 1.918 | 3.633 | 7895 | 14967 |
| PbCT | 16066 | 1.101 | 2.427 | 32121 | Dominated |
| PfCT | 14780 | 1.061 | 2.607 | Reference | Dominated |

Abbreviation: PbCT: pemetrexed based chemotherapy; PfCT: pemetrexed free chemotherapy; LY: life year; QALY: quality-adjusted life year; ICER: incremental cost-effectiveness ratio.

^a^compared with PfCT; ^b^sequential ICER was compared with the next best non-dominated option.

**eTable 9.** Scenario 2: summary of cost-effectiveness results in the UK (utility for PFS and PD).

| **Treatments** | **Costs (£)** | **QALYs** | **LYs** | **ICER^a^**  **(£/QALY)** | **sequential ICER^b^ (£/QALY)** |
| --- | --- | --- | --- | --- | --- |
| Gefitinib | 24529 | 1.409 | 2.571 | -3349 | Dominant |
| Osimertinib | 139483 | 1.976 | 3.485 | 161815 | Dominated |
| Afatinib | 51865 | 1.575 | 2.899 | 87665 | Dominated |
| Dacomitinib | 155510 | 1.717 | 3.080 | 291197 | Dominated |
| Erlotinib | 27237 | 1.413 | 2.554 | 15550 | Extended dominated |
| Afatinib+Cetuximab | 92935 | 1.372 | 2.535 | 659016 | Dominated |
| Erlotinib+Bevacizumab | 106486 | 1.676 | 2.950 | 199989 | Dominated |
| Gefitinib+Pemetrexed | 33221 | 1.696 | 3.080 | 19255 | 30331 |
| Gefitinib+PbCT | 44445 | 1.978 | 3.632 | 27424 | 39791 |
| PbCT | 31595 | 1.256 | 2.426 | -524122 | Dominated |
| PfCT | 24999 | 1.269 | 2.607 | Reference | Dominated |

Abbreviation: PbCT: pemetrexed based chemotherapy; PfCT: pemetrexed free chemotherapy; LY: life year; QALY: quality-adjusted life year; ICER: incremental cost-effectiveness ratio.

^a^compared with PfCT; ^b^sequential ICER was compared with the next best non-dominated option.

**eTable 10.** Scenario 2: summary of cost-effectiveness results in China (utility for PFS and PD).

| **Treatments** | **Costs (£)** | **QALYs** | **LYs** | **ICER^a^**  **(£/QALY)** | **sequential ICER^b^ (£/QALY)** |
| --- | --- | --- | --- | --- | --- |
| Gefitinib | 12961 | 1.875 | 2.572 | -33322 | Dominant |
| Osimertinib | 25459 | 2.573 | 3.485 | 14205 | Dominated |
| Afatinib | 21478 | 2.110 | 2.900 | 23194 | Dominated |
| Icotinib | 18308 | 1.809 | 2.482 | -287483 | Dominated |
| Dacomitinib | 22517 | 2.260 | 3.081 | 17621 | Dominated |
| Erlotinib | 19270 | 1.868 | 2.554 | 95282 | Dominated |
| Afatinib+Cetuximab | 52380 | 1.841 | 2.536 | 1867757 | Dominated |
| Erlotinib+Bevacizumab | 58504 | 2.176 | 2.951 | 122961 | Dominated |
| Gefitinib+Pemetrexed | 16873 | 2.251 | 3.081 | 4865 | 10413 |
| Gefitinib+PbCT | 21545 | 2.634 | 3.633 | 8319 | 12200 |
| PbCT | 16066 | 1.736 | 2.427 | -15076 | Dominated |
| PfCT | 14780 | 1.821 | 2.607 | Reference | Dominated |

Abbreviation: PbCT: pemetrexed based chemotherapy; PfCT: pemetrexed free chemotherapy; LY: life year; QALY: quality-adjusted life year; ICER: incremental cost-effectiveness ratio.

^a^compared with PfCT; ^b^sequential ICER was compared with the next best non-dominated option.

**eTable 11.** Scenario 3: summary of cost-effectiveness results in the UK (disutility for SAE).

| **Treatments** | **Costs (£)** | **QALYs** | **LYs** | **ICER^a^**  **(£/QALY)** | **sequential ICER^b^ (£/QALY)** |
| --- | --- | --- | --- | --- | --- |
| Gefitinib | 24529 | 1.134 | 2.571 | -1365 | Dominant |
| Osimertinib | 139483 | 1.765 | 3.485 | 117375 | 2189970 |
| Afatinib | 51865 | 1.268 | 2.899 | 56079 | Dominated |
| Dacomitinib | 155510 | 1.484 | 3.080 | 187809 | Dominated |
| Erlotinib | 27237 | 1.182 | 2.554 | 5696 | Extended dominated |
| Afatinib+Cetuximab | 92935 | 1.136 | 2.535 | 196126 | Dominated |
| Erlotinib+Bevacizumab | 106486 | 1.551 | 2.950 | 107005 | Dominated |
| Gefitinib+Pemetrexed | 33221 | 1.411 | 3.080 | 13231 | 31410 |
| Gefitinib+PbCT | 44445 | 1.721 | 3.632 | 20864 | 36138 |
| PbCT | 31595 | 0.880 | 2.426 | 72441 | Dominated |
| PfCT | 24999 | 0.789 | 2.607 | Reference | Dominated |

Abbreviation: PbCT: pemetrexed based chemotherapy; PfCT: pemetrexed free chemotherapy; LY: life year; QALY: quality-adjusted life year; ICER: incremental cost-effectiveness ratio.

^a^compared with PfCT; ^b^sequential ICER was compared with the next best non-dominated option.

**eTable 12.** Scenario 3: summary of cost-effectiveness results in China (disutility for SAE).

| **Treatments** | **Costs (£)** | **QALYs** | **LYs** | **ICER^a^**  **(£/QALY)** | **sequential ICER^b^ (£/QALY)** |
| --- | --- | --- | --- | --- | --- |
| Gefitinib | 12961 | 1.312 | 2.572 | -7641 | Dominant |
| Osimertinib | 25459 | 1.936 | 3.485 | 12396 | 512331 |
| Afatinib | 21478 | 1.472 | 2.900 | 16837 | Dominated |
| Icotinib | 18308 | 1.254 | 2.482 | 19642 | Dominated |
| Dacomitinib | 22517 | 1.658 | 3.081 | 13251 | Dominated |
| Erlotinib | 19270 | 1.342 | 2.554 | 16786 | Dominated |
| Afatinib+Cetuximab | 52380 | 1.305 | 2.536 | 163196 | Dominated |
| Erlotinib+Bevacizumab | 58504 | 1.677 | 2.951 | 72576 | Dominated |
| Gefitinib+Pemetrexed | 16873 | 1.607 | 3.081 | 3926 | 13258 |
| Gefitinib+PbCT | 21545 | 1.928 | 3.633 | 7923 | 14564 |
| PbCT | 16066 | 1.105 | 2.427 | 41274 | Dominated |
| PfCT | 14780 | 1.074 | 2.607 | Reference | Dominated |

Abbreviation: PbCT: pemetrexed based chemotherapy; PfCT: pemetrexed free chemotherapy; LY: life year; QALY: quality-adjusted life year; ICER: incremental cost-effectiveness ratio.

^a^compared with PfCT; ^b^sequential ICER was compared with the next best non-dominated option.

**eTable 13.** Scenario 4: summary of cost-effectiveness results in the UK (drug acquisition costs from British national formulary).

| **Treatments** | **Costs (£)** | **QALYs** | **LYs** | **ICER^a^**  **(£/QALY)** | **sequential ICER^b^ (£/QALY)** |
| --- | --- | --- | --- | --- | --- |
| Gefitinib | 44521 | 1.130 | 2.571 | 41588 | Dominated |
| Osimertinib | 139607 | 1.762 | 3.485 | 109152 | 813813 |
| Afatinib | 51993 | 1.255 | 2.899 | 46084 | Extended dominated |
| Dacomitinib | 155635 | 1.475 | 3.080 | 173902 | Dominated |
| Erlotinib | 34473 | 1.177 | 2.554 | 13927 | Dominant |
| Afatinib+Cetuximab | 93061 | 1.113 | 2.535 | 175119 | Dominated |
| Erlotinib+Bevacizumab | 116654 | 1.541 | 2.950 | 110684 | Dominated |
| Gefitinib+Pemetrexed | 59428 | 1.401 | 3.080 | 47160 | Extended dominated |
| Gefitinib+PbCT | 78571 | 1.687 | 3.632 | 53128 | 86467 |
| PbCT | 32738 | 0.861 | 2.426 | 36574 | Extended dominated |
| PfCT | 28442 | 0.744 | 2.607 | Reference | Extended dominated |

Abbreviation: PbCT: pemetrexed based chemotherapy; PfCT: pemetrexed free chemotherapy; LY: life year; QALY: quality-adjusted life year; ICER: incremental cost-effectiveness ratio.

^a^compared with PfCT; ^b^sequential ICER was compared with the next best non-dominated option.

**eTable 14.** Scenario 5: summary of cost-effectiveness results in the UK (osimertinib included in subsequent therapy).

| **Treatments** | **Costs (£)** | **QALYs** | **LYs** | **ICER^a^**  **(£/QALY)** | **sequential ICER^b^ (£/QALY)** |
| --- | --- | --- | --- | --- | --- |
| Gefitinib | 28696 | 1.130 | 2.571 | -3184 | Dominant |
| Osimertinib | 139483 | 1.762 | 3.485 | 107573 | 1209143 |
| Afatinib | 56095 | 1.255 | 2.899 | 51205 | Dominated |
| Dacomitinib | 159611 | 1.475 | 3.080 | 177308 | Dominated |
| Erlotinib | 31323 | 1.177 | 2.554 | 3224 | Extended dominated |
| Afatinib+Cetuximab | 97065 | 1.113 | 2.535 | 181945 | Dominated |
| Erlotinib+Bevacizumab | 110402 | 1.541 | 2.950 | 100976 | Dominated |
| Gefitinib+Pemetrexed | 37729 | 1.401 | 3.080 | 11875 | 33399 |
| Gefitinib+PbCT | 48921 | 1.687 | 3.632 | 20131 | 39070 |
| PbCT | 36322 | 0.861 | 2.426 | 54447 | Dominated |
| PfCT | 29927 | 0.744 | 2.607 | Reference | Dominated |

Abbreviation: PbCT: pemetrexed based chemotherapy; PfCT: pemetrexed free chemotherapy; LY: life year; QALY: quality-adjusted life year; ICER: incremental cost-effectiveness ratio.

^a^compared with PfCT; ^b^sequential ICER was compared with the next best non-dominated option.

**eTable 15.** Scenario 5: summary of cost-effectiveness results in China (osimertinib included in subsequent therapy).

| **Treatments** | **Costs (£)** | **QALYs** | **LYs** | **ICER^a^**  **(£/QALY)** | **sequential ICER^b^ (£/QALY)** |
| --- | --- | --- | --- | --- | --- |
| Gefitinib | 13079 | 1.312 | 2.572 | -7614 | Dominant |
| Osimertinib | 25459 | 1.935 | 3.485 | 11980 | 214664 |
| Afatinib | 21600 | 1.469 | 2.900 | 16215 | Dominated |
| Icotinib | 18427 | 1.254 | 2.482 | 17858 | Dominated |
| Dacomitinib | 22633 | 1.656 | 3.081 | 12860 | Dominated |
| Erlotinib | 19384 | 1.340 | 2.554 | 15790 | Dominated |
| Afatinib+Cetuximab | 52496 | 1.297 | 2.536 | 158920 | Dominated |
| Erlotinib+Bevacizumab | 58612 | 1.673 | 2.951 | 71368 | Dominated |
| Gefitinib+Pemetrexed | 17061 | 1.606 | 3.081 | 3810 | 13526 |
| Gefitinib+PbCT | 1.918 | 3.633 | 7873 | 1.918 | 14965 |
| PbCT | 16262 | 1.101 | 2.427 | 31897 | Dominated |
| PfCT | 14985 | 1.061 | 2.607 | Reference | Dominated |

Abbreviation: PbCT: pemetrexed based chemotherapy; PfCT: pemetrexed free chemotherapy; LY: life year; QALY: quality-adjusted life year; ICER: incremental cost-effectiveness ratio.

^a^compared with PfCT; ^b^sequential ICER was compared with the next best non-dominated option.
